# The face of war: Trauma analysis of a mass grave from the Battle of Lützen (1632)

**DOI:** 10.1371/journal.pone.0178252

**Published:** 2017-05-22

**Authors:** Nicole Nicklisch, Frank Ramsthaler, Harald Meller, Susanne Friederich, Kurt W. Alt

**Affiliations:** 1 State Office for Heritage Management and Archaeology Saxony-Anhalt – State Museum of Prehistory, Halle (Saale), Germany; 2 Danube Private University, Krems-Stein, Austria; 3 Institute of Legal Medicine, Saarland University, Homburg (Saar), Germany; University of Otago, NEW ZEALAND

## Abstract

Contemporary accounts of battles are often incomplete or even erroneous because they reflect the—often biased—viewpoints of the authors. Battlefield archaeology faces the task of compiling an historical analysis of a battle and of gathering all the available facts. Besides cultural historical evidence and artefacts, the human remains of those who have fallen in battle also provide invaluable information. In studying mass graves from a military context, the injury types and patterns are significant. They allow us to reconstruct the circumstances surrounding the soldiers’ deaths and provide information on the hostilities that occurred on the battlefield. One such mass grave was discovered in 2011 at Lützen, Saxony-Anhalt (Germany). Based on its geographical location and on the results obtained from archaeological examinations carried out in the area, the grave could be dated to the Thirty Years War (1618–1648). Further archaeological research confirmed that the dead had been soldiers from the Battle of Lützen (1632). The mass grave was block-lifted and then comprehensively examined at the State Museum of Prehistory in Halle (Saale). As well as osteological examinations to determine age, sex, height, state of health, i.e. diseases or injuries, imaging methods were also employed and histological and isotopic analyses carried out. The focus of this study was on the injuries sustained by the soldiers both prior to and during the battle. The results revealed that the 47 deceased had been between the ages of 15 and 50 when they died. Numerous healed injuries showed that the men had often been involved in violent encounters. Approximately three in every four soldiers had injuries that could have been fatal. Wounds inflicted by handguns, particularly to the skull, were predominant. The integrative analysis of the archaeological and anthropological data allowed us to conclude that the majority had been killed during a cavalry attack.

## Introduction

Religious schism and disputes over political power within Europe created discord which led to the Thirty Years War, sparked by the Defenestration of Prague on 23^rd^ May 1618 [1,2]. On that occasion a number of Catholic Lords Regent were thrown out of the window and into the castle moat by the mainly Protestant assembly members. Thus began the Thirty Years War. Under the pretext of religious interests, the very foundations of the Holy Roman Empire would be shaken, and this would manifest itself in numerous military campaigns until 1648. The Danish King Christian IV (1577–1648) and Gustavus II Adolphus King of Sweden (1597–1632), who were both Protestants, felt duty-bound to stand with their fellow believers and eventually became involved in the conflict. In 1630, King Gustavus II Adolphus crossed over to the mainland, where he fought a succession of victorious battles [1,2]. It was not until the autumn of 1632 after an undecided clash near Nuremberg, that he lost his reputation for invincibility. Not long after that, on 6^th^ or 16^th^ November he once again faced his archenemy General Albrecht von Wallenstein (1583–1634), commander of the imperial troops, at Lützen [2,3]. This battle would have the gravest consequences, both for Sweden and for the whole of Europe.

According to the Gregorian calendar still in use today, the Battle of Lützen took place on 16^th^ November 1632. The alternative date stems from the fact that Pope Gregory XIII ordered the switch from the Julian to the Gregorian calendar in 1582, resulting in 10 days being skipped. The Holy Roman Empire was very quick to adopt the new calendar, whilst some of the Protestant countries rejected it and retained the old calendar for another 300 years. Whilst Scandinavian countries also eventually switched to the Gregorian calendar, the Battle of Lützen is still to this day dated according to the old calendar, which once again illustrates the lasting pain felt by the nation due to the loss of King Gustavus II Adolphus, who was mortally wounded on the battlefield [4]. Besides the royal leader, between 6000 and 9000 soldiers also lost their lives [4,5]. There is still debate as to who actually won the battle [2,6]. The fact that Wallenstein was first to order his troops to retreat would argue in favour of a Swedish victory. However, the death of the King of Sweden was a significant factor and the imperial army had clearly captured more Swedish standards. From a neutral perspective there were no victors and the war would continue for another 16 years.

### Being a soldier in the 17^th^ century

The climatic conditions in the 17^th^ century were characterised by the Little Ice Age [7,8]. Consecutive long cold winters and cool wet summers had led to crop failures and food shortages in the Late Middle Ages and the early post-medieval period and the population had become more and more discontent. Due to prevalent infectious diseases such as tuberculosis, typhoid and most importantly various outbreaks of the plague, the population had been considerably reduced [9–11]. The battles, sieges, looting and destruction of agricultural land during the war made life even harder for the rural population.

Against this background, there were many reasons to “voluntarily” enter military service [12–14]. Numerous recruits were driven by hunger and poverty, whilst others went in search of wealth or fame and glory. Only a very small minority were fighting for ideals or religious convictions. Beggars, vagrants and criminals could be forcibly recruited [12] and conscription had already been introduced in Sweden and Finland. Each province and parish had to recruit a certain number of men, but actually most soldiers were recruited in foreign countries such as Germany. Desertion was a major problem and, besides losses due to illness and injury, had an added impact on troop numbers [12,13,15,16].

Whilst a soldier’s pay and rations were supposed to be incentives to join the army, frequent disruption to supplies and overdue wages, however, drove the soldiers to looting which, unfortunately for the civilian population, was often quite violent [12,14,15]. A soldier’s life was hard and the supply situation fluctuated constantly [15,17]. This is also clear from the autobiographical account of Peter Hagendorf, a German mercenary soldier [18]. Phases of sufficient supplies could soon be followed by misery and hardship. It was possible for mercenaries to change sides so that today’s ally could become tomorrow’s enemy.

It is assumed that the men often suffered from significant physical and psychological stress [14,16]. Violence, cruelty and death were ever-present, not only on the battlefield but also in a soldier’s everyday life. Stimulants such as spirits and tobacco were extremely popular at the time [16,19].

### Battlefield archaeology

In contrast to other countries such as Great Britain (e.g. [20–25]), Sweden (e.g. [26,27]) and the US (e.g. [28,29]), German battlefield archaeology is still in its infancy [30–35]. Whilst conflict archaeology has already gained widespread acceptance in prehistoric research (e.g. [36,37]), the benefit of examining post-medieval sites is still often questioned. Such resistance and scepticism may be rooted in, among other things, Germany’s disastrous military past. Conflict and war, however, are aspects of human culture and archaeological research into our recent history is of crucial importance for future generations and their consciousness of peace.

Battlefield archaeology provides important clues with regard to the localisation of battlefields and the course of individual battles, thereby adding to historical reconstructions primarily based on written sources and drawings, whose descriptions are rarely objective. In the past 10 years, however, interest in and recognition of bioarchaeological research carried out on post-medieval theatres of war has considerably increased (e.g. [38–46]).

Besides the material finds that were recovered during surveys of the Lützen battlefield, the mass grave discovered in 2011 illustrates in a very tragic way what was left after a day of battle. Examining the human remains provides us with an opportunity to reconstruct the violent events that took place on the battlefield on the one hand and to gain an insight into the living conditions of the soldiers on the other. The grave and the bioarchaeological analyses were components of an exhibition and preliminary results have already been published in the accompanying volume of the exhibition [45]. The main aim of this study was to analyse the fatal injuries the men sustained during the battle. Examining the unhealed traumata offered clues concerning the fighting and the military and strategic operations on the battlefield. Healed injuries from previous confrontations were also recorded and studied, since many of the men had been in mercenary service since the beginning of the war. Evidence from other mass graves of that period, such as Wittstock (1636, [40,47,48]), Neubrandenburg (1631, [49]) and Alerheim (1645, [41]), was included in the interpretation and discussion of the results. However, more recent features from the 18^th^ century such as the mass grave at Stralsund (1715, [50]) and the examinations carried out by Cooper [51] on mass graves in Switzerland (1799/1800) as well as earlier features including the medieval mass grave at Towton (1461) and the 16^th^ century mass graves at Uppsala (c. 1520) and Alkmaar (1573) are also good examples for comparison [21,52,53].

## Materials and methods

This study deals with archaeological skeletal material whose excavation and scientific examination was commissioned and licensed by the State Office for Heritage Management and Archaeology Saxony-Anhalt (under the directorship of Prof. Dr. H. Meller). The human remains (Activity-No. 1946/102) are stored in the archive of the State Office for Heritage Management and Archaeology Saxony-Anhalt at Halle (Saale). The inventory list of skeletons corresponds with the information given in Table 1 and the supporting information.

**Table 1:** Individual data obtained from the age, sex and trauma analysis.

ID	Age range (years)	Sex	Cranial injuries				Postcranial injuries				Probable cause of death
			projectile	blunt	sharp	antem.	projectile	blunt	sharp	antem.	
I1	26–35	M	n.d	n.d	n.d	n.d	0	0	2	0	sharp force
I2	30–40	M	0	1	0	1 (b/p)	2	0	0	1 (b/p)	projectile trauma
I3	19–26	M	1	0	0	0	0	0	0	0	projectile trauma
I4	19–26	M	0	1	0	0	0	0	0	1 (b)	blunt force?
I5	30–40	M	1	0	0	1 (b)	0	0	0	0	projectile trauma
I6	19–26	M	1	0	0	0	0	0	0	1 (s)	projectile trauma
I7	24–30	M	1	0	0	1 (b)	0	0	1	0	projectile trauma
I8	35–45	M	1	0	0	0	0	0/1	0	0	projectile trauma
I9	15–19	M	0	0/1	4	1 (b)	1	0	0	0	sharp force
I10	15–18	M?	0	1	0	0	0	0	0	0	blunt force?
I11	24–30	M	0	0	0	0	0	1	3	0	sharp force
I12	30–40	M	1	1	0	1 (s)	0	0	0	0	projectile trauma
I13	25–30	M	0	1	0	0	0	1	1	1 (b)	sharp force
I14	25–35	M	1	0	0	1 (b)	0	0	0	0	projectile trauma
I15	30–40	M	1	0	0	1 (b)	0	1	0/1	3 (b)	projectile trauma
I16	25–35	M	0	1	0	0	2	0	0	0	projectile trauma
I17	19–26	M	0	0/2	0	0	0	0	0	3 (b+s)	blunt force?
I18	40–50	M	1	1	0	1 (b)	0	0	0	1 (b)	projectile trauma
I19	35–45	M	0	0	0	0	0	0	0	1 (b)	n.d.
I20	19–26	M	0	0/1	0	0	0	0	0	0	n.d.
I21	24–30	M?	0	0	0	0	0	0	0/1	0	sharp force?
I22	19–26	M?	1	0	0	0	0/2	0	0	0	projectile trauma
I23	24–30	M	0	1	0	4 (s)	0	0	0	3 (b+st)	n.d.
I24	16–20	M	0	0	0	0	0	0	0	1 (st)	n.d.
I25	30–40	M?	0	0/1	0	0	0	0/1	0	0	n.d.
I26	25–35	M	1	0	0	0	0	0	1	1 (b)	projectile trauma
I27	30–40	M?	0	0/1	0	0	0	0	0	0	n.d.
I28	19–26	M	0	0/1	0	0	0	1/2	0	0	blunt force?
I29	19–26	M	1	0	0	0	0	0	0	0	projectile trauma
I30	18–25	M?	1	0	0	0	0	0	0	1 (b/s)	projectile trauma
I31	24–30	M	1	0	0	0	0	0	0	0	projectile trauma
I32	14–16	M?	0	1	0	0	0	0	0	0	blunt force?
I33	19–26	M	0/1	0	0	0	1	0	2	1 (s)	projectile trauma?
I34	24–30	M	1	1	0	1 (b/p)	1	0	0	1 (s/p)	projectile trauma
I35	19–26	M?	1	0	1	0	0	0	0	0	projectile trauma
I36	19–26	M?	0	0	0	0	0	0	0/1	1 (b)	n.d.
I37	26–35	M	1	0	0	0	0	0	0/1	4 (b+s/p)	projectile trauma
I38	30–40	M	0	1	0	0	0	0	0	0	n.d.
I39	26–35	M	2	0	0	0	0	0	0	1 (b)	projectile trauma
I40	30–40	M	1	0	0	0	0	0	0	1 (b)	projectile trauma
I41	19–26	M	0	0	0	0	1	0	1	0	projectile trauma
I42	40–50	M?	1	0	0	0	0	1	0	0	projectile trauma
I43	24–30	M	0	0	0	1 (s)	1	0	0	1 (b)	projectile trauma?
I44	26–35	M?	0/1	2	0	0	0	0	0	0	cranial injury
I45	19–26	M	0	0	0	0	0	3	0	0	n.d.
I46	24–30	M	0	0	0	2 (b+s)	1	0	0	1 (s)	projectile trauma
I47	24–30	M	1	0	0	0	0	0	0	1 (b)	projectile trauma
	n injuries (reliable)		22	13	5	16	10	8	11	30	
	n injuries (probable)		2	7	---	---	2	4	4	---	

antem. = antemortem; b = blunt; s = sharp; p = projectile; st = stress fracture; reliable/probable; (either/or); n.d. = not determinable; subj. = subjective; M? = probable Male

**Fig 1:**
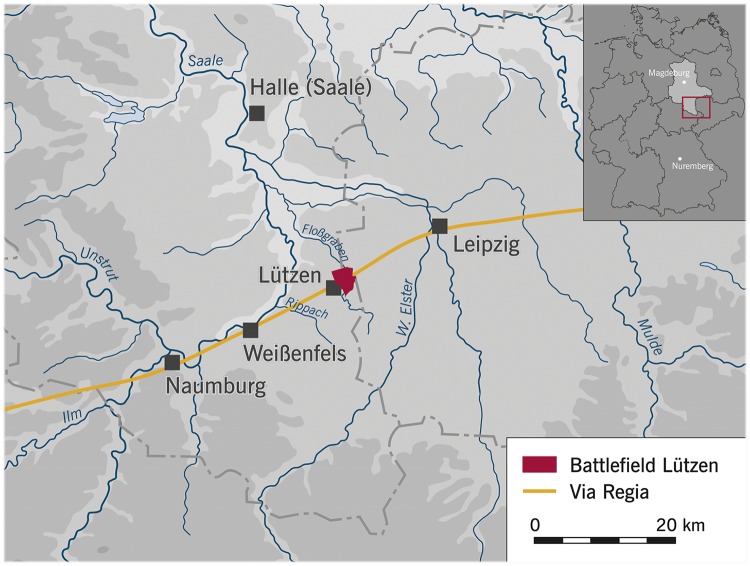
Map of the site and its surroundings (LDA Halle/Saale).

The battlefield of 1632 covered an area of approximately 750 hectares. Beginning in September 2006, the first large-scale archaeological examinations by the State Office for Heritage Management and Archaeology Saxony-Anhalt were undertaken using metal detectors, which ultimately brought to light approximately 3000 metal objects from the battle [44,54]. An analysis of the distribution of the projectiles allowed the archaeologists to reconstruct the hostilities and to draw conclusions with regard to the course of the battle [44,55]. Neither aerial photography nor geophysical examinations (geomagnetic and ground-penetrating radar) provided any evidence of graves [54]. The records kept by the city authorities of Lützen, however, showed that construction work had repeatedly unearthed bone finds on the north-eastern edge of the city. In the late summer of 2011, a linear trial trench, excavated parallel to an old trade route (*Via Regia*) with the aim of verifying data obtained by metal detector surveying, led to the chance discovery of a mass grave (Fig 1).

Originally the pit measured approximately 3.5 x 4.6 m and had been dug to a depth of c. 2.5–3 m, at which point the high groundwater level prevented any further digging [54]. Attracted by the military remains that might come to light, illicit excavations began to be carried out right after the discovery, prompting immediate intervention on the part of the archaeological services. Due to time constraints and safety issues and in view of the impending poor weather conditions, it would not have been possible to secure and expose the remains on site using the necessary detailed stratigraphic and taphonomic excavation methods, which made block lifting seem like the only appropriate option. Given the flat rectangular shape of the mass grave and to ensure compliance with environmental law due to the location of the site near a central regional drinking fountain, the block, which was very brittle because of its size, had to be stabilised in order to prevent certain sections from breaking off during recovery. According to static and geotechnical calculations this could only be accomplished by cutting the block in two. In order to avoid losing any of the monument’s informative value, this was achieved by means of a wire saw, resulting in a loss of approximately two to three centimetres of material. Both blocks (each weighing c. 25 t) were brought by heavy goods vehicle to the State Museum of Prehistory in Halle (Saale), where the skeletons were excavated and studied [54].

Once the topmost layer had been exposed, the overall impression of the grave was the decisive factor in the decision to preserve the block as a whole, which would most certainly be open to discussion. The recovery and archiving of skeletal remains, mummies or bog bodies has justifiably prompted fundamental debate as to whether it is ethical to recover human bodies, carry out invasive examinations and even present them to the public or whether this constitutes a violation of the personal rights of the deceased [56]. Should biohistorical sources be used in museums and in the media as exhibits? Both sides of the argument have considerable support and the decision is never easy or free of conflict. Each case must be judged on its individual merits.

In an excavation, the individuals or bones are usually recovered and then stored in boxes. In this case, however, a different approach was taken because we felt that the Lützen mass grave constituted an impressive overall monument of the Thirty Years War and of war in general—it is a representation of war in all its cruelty and, unfortunately, shocking topicality. By coincidence, or perhaps intentionally, the last body (indiv. 13) placed in the grave was lying in a different position to the other individuals, in a cruciform pose on top of the other deceased. This crucial aspect of the overall impression would have been lost if the usual method of “dissecting” the block had been applied. The exhibition “Krieg—eine archäologische Spurensuche” (War—an archaeological search for evidence; end of 2015 to mid-2016) with the block as its centrepiece was seen by more than 58,000 visitors and very positively received. Negative connotations in terms of ethical principles largely failed to materialise and the immediate authentic potency of the grave and the link to the scientific explanations was recognised; this had never previously been achieved to such an extent by exhibiting 3D-prints or other types of displays. In our view, the manner of confronting visitors with the reality of war while still maintaining an abstract and scientifically underpinned form did, in fact, do justice to the archaeological context on one hand and to the Lützen mass grave as a monument against war on the other. However, it does continue to spark debate.

The osteological examination of the skeletons was largely carried out *in situ*. Both block burials were first excavated from the top, samples (bones and teeth) were taken for biochemical and molecular genetic analyses and the skeletons were osteologically examined. The blocks were then turned over and the skeletons exposed and examined from the underside. Some of the bones were temporarily removed for more detailed osteological analysis, for instance long bones (femora) were used to estimate body height or skulls were examined for suspected perimortem injuries. Rib samples were taken for absolute chronological validation by radiocarbon dating at the Curt-Engelhorn-Centre for Archaeometry in Mannheim.

### Age and sex

Age and sex of the deceased were identified using as comprehensive a repertory of morphognostic methods as possible (cf. [57,58]; S2 Table). However, due to the examination *in situ*, the options of analysis were limited. Sex determination was carried out mainly by studying the classical features of the pelvis and skull [58–61] although general impressions of robustness such as the size and formation of the joints and muscle attachment sites were also considered in the diagnosis. Morphological criteria, particularly of the mandible and ilium, also deliver valuable information for the identification of adolescent individuals and are even recommended for much younger age groups [62]. However, caution should be exercised since skeletal maturation is still incomplete and the expression of sex-related features is highly variable; while some individuals exhibit distinct features others may be indeterminable.

Cranial suture obliteration, dental wear and features of the pelvis (pubic symphysis and auricular surface) were analysed in order to determine age at death [61,63–66]. Since features of the pelvis hold the promise of greater accuracy, more weight was given to them in the age diagnoses but all available criteria were taken into account to improve the accuracy of determination. In the younger individuals from the mass grave, whose skeletons were still in development, the degree of ossification or rather the epi- and apophyseal union on the postcranial bones was identified (including the clavicle, scapula, pelvis and long bones) [61]. Moreover, the formation and eruption of the teeth (molars in particular) were taken into account [67].

### Palaeopathological examination and trauma analysis

In examinations of mass graves, the identification of perimortem injuries, besides age and sex of the dead, also plays an important role in the interpretation of the overall feature, since (natural) catastrophes or epidemics can also lead to large numbers of bodies being buried simultaneously [68–70]. In cases where the mass grave is linked to an historically documented battle, information regarding injury types and patterns can assist in reconstructing the course of the battle and the nature of the specific warfare (e.g. [40,52,71]). Healed injuries provide information on the lives and suffering of the individuals and on the medical care they received.

The traumatic changes to the bones were studied macroscopically by using a magnifying glass and scattered light. In some cases, bones were removed from the grave for more in-depth study by X-Ray, CT, histology or osteometric analyses. Because the skeletons largely remained *in situ*, it was not possible to collect a complete palaeopathological dataset. Only the prominent features could be documented whilst others such as joint status, muscle marks and anatomical variants could not be (serially) studied in greater detail or changes quantified. For this reason, it was difficult to calculate true prevalence rates for the injuries. However, calculations of detailed prevalence rates can be found in the supplement.

Forensic science distinguishes between blunt and sharp force traumata and gunshot wounds, which have been studied both in recent victims of accidents and violence and in the remains recovered from archaeological contexts [72–81]. The observations made regarding the dead from the Lützen mass grave were analysed on the scientific basis of standardised procedures.

#### Blunt force trauma

Blunt force trauma is basically caused by a body colliding with a hard, flat or blunted surface [72,75,78,79,82]. Compression, shearing and bending forces come into play, impacting on the bone and causing injuries ranging from simple two-segmented fractures (e.g. transverse fractures) to complex comminuted fractures. This can be due to a blow from an object and therefore direct trauma (e.g. “parry” fracture). However, a fall or an attempt at absorbing a fall can also lead to characteristic lesions (e.g. Colles’ fracture). In an archaeological context, it can be extremely difficult to distinguish between trauma inflicted intentionally and trauma caused by an accidental event, particularly in injuries to the torso and the extremities [83]. Cranial trauma seems to be of special interest because interpersonal violence is often directed at the head as the location of the victim’s identity [84]. In analysing cranial injuries, attention should be paid to typical fracture patterns with often discriminable rounded and angled fracture lines as well as penetrating injuries with bevelled openings. Deformations of the skull can either impact on the *tabula externa* only (depression, incomplete fracture) or they can also involve the *tabula interna* (impression, complete fracture) [85,86]. Indentations in the cranial vault can be interpreted as the remnants of healed depression or impression fractures. Besides, an impact may also result in a penetrating fracture defect where a fragment of bone has been detached completely and displaced. Certain objects/weapons can leave behind characteristically shaped imprints on the bone allowing them to be identified [78]. The force of the impact determines the degree of fragmentation. To put it simply, force applied slowly more often results in incomplete fractures with deformations, while rapid force causes complete penetrating fractures with little or no deformation [78,79,87]. These criteria can also be used to distinguish between blunt force and projectile trauma. Galloway and colleagues [87] stated that “…, as the speed of the applied force increases, such as in the case of high-velocity projectile impacts, bone's ability to absorb energy increases. When the bone fails, however, it reacts as a brittle material and the resulting fracture damage is catastrophic. In this scenario, the conjoining fractures fit together cleanly, and there is little to no plastic deformation of the bone fragments. Alternatively, with a slowly applied force, bone reacts as a viscoelastic material and, prior to failure, will deform in shape to accommodate the stress” ([87], p. 57). However, factors that may complicate matters include the magnitude of the force, the size of the area impacted and the thickness of the bone affected. The point of impact may be identified by radiating fracture lines which move outward in any direction following the weakest areas of the bone [78,79]. In addition, concentric lines can develop between the radial lines, thus outlining the point of impact. Sutures and pre-existing fractures terminate fracture lines, which enables sequencing of multiple impacts (so-called Puppe`s law) [79].

#### Sharp force trauma

Sharp force results in cutting, stabbing and chopping traces such as those left behind by sharp knives, axes or swords [78,79,82]. Such injuries have straight lines and relatively smooth surfaces and, depending on the shape of the blade, a more or less V-shaped profile. In examining such evidence, attention should also be paid to the width, depth and length of the injury and to striations or grooves on the margins of the wound. Fracture lines can radiate outwards from the primary blow or penetration site and bone fragments can be detached completely. A detailed description of the wound not only contributes to the recognition of sharp force injuries but may also help identify the weapon involved [79]. A further important point is that the impact of an axe or mattock can create fracture patterns similar to those caused by blunt force (chop wounds) [75,77]. Thus, it can be difficult to distinguish between these injuries and those caused by blunt edges. In cases of sharp force injuries, special attention should be paid to possible defence injuries to the hands or forearms such as cuts, stab wounds or factures.

#### Projectile trauma

Strictly, projectile (ballistic) wounds are a special type of blunt force trauma [82]. Wounds inflicted by arrows or spears can also be classed as projectile or ballistic injuries. In identifying projectile trauma on bone attention must be paid to the margins of the wound which, particularly on the skull, will exhibit bevelling around the impact site [88]. Its shape and size provide information on the angle of impact, the size and the kinetic energy of the projectile [78,79,89,90]. The fracture zone in the area of the exit wound can be larger and more irregularly shaped than the entry wound, particularly in cases where the projectile deformed when it was fired or on impact. A bullet entering a bone at right angles usually causes a round bullet hole. If the impact angle changes, oval or keyhole-shaped wounds can also occur. Due to the hydraulic pressure that occurs inside the skull radial (secondary burst fractures) and concentric (tertiary bending fractures) fracture lines can develop [79,89,91].

The effect of a projectile is dependent on several variables. Besides form, material, weight and calibre of the projectile, other factors such as its impact velocity, deviations from its trajectory, the distance it has travelled, the angle at which it penetrates the bone and the gunpowder used [92–95] also play a role. Round lead bullets were generally in use in the 17^th^ century and depending on the type of weapon these could exhibit great variability in size and weight [92,95]. The calibre refers to the diameter of the projectile.

The detailed examination of the projectile injuries involved the measuring and weighing of the bullets found in the mass grave and recording whether they were deformed or not. Bullets with slight deformations could be categorised as pistol, carbine or musket balls using contemporary books on weaponry and weapons collections [95,96]. However, due to overlapping amongst the calibres, it is not always possible to determine from which type of firearm a bullet came.

Moreover, in some cases it can be difficult to reconstruct the angle of the shot, because the trajectory of the projectile can be bent, particularly in the skull (internal ricochet), causing the entry and exit wounds to not necessarily lie in a straight line [91,97]. The extent of the damage also depends on anatomical structures such as cranial sutures or the structural buttresses of the skull (trajectories) [72,93]. Thanks to their higher density, projectiles or lead fragments left behind in the body or bone can be localised using CT scans (e.g. [98]).

#### Timing of bone damage

Damage to the bone can have occurred during a person’s lifetime (antemortem), sometime after death (postmortem) or around the time of death (perimortem). It is therefore important when interpreting injuries to determine at what point in time the damage was inflicted [76,78,81,84,87,99,100]. Injuries that occurred during a person’s lifetime and which they survived can be identified beyond doubt thanks to traces of healing. The duration of the healing varies but one would generally expect a bone to show clear signs of new formation and remodelling after approximately one month [99]. If there are no signs of healing, this might point to a perimortem injury, although it is important to distinguish between perimortem and postmortem damage, which can be difficult, particularly where the bone is poorly preserved. Important features are the course of the fracture lines and the colour of the fracture edges because a “fresh” bone which is still rich in fat and collagen breaks in a different way to a “dry” bone, which has already begun to decay [90,99–101]. Attention must also be paid to gnaw marks from carnivores or rodents which can lead to pathological misdiagnoses [102,103].

#### Imaging techniques

Conventional radiographs of pathological changes to the bones from the Lützen mass grave were taken using an analogue X-ray apparatus at the State Museum of Prehistory in Halle (Saale). Computed tomography (CT) scans were taken of some of the skulls at the University Clinic in Halle (Saale). The data were processed using the computer software OsiriX.

## Results

### The grave

The feature was dated primarily on the basis of its geographical location and the results from the archaeological examination of the area (surveys), based upon which the mass grave can be associated with the battlefield of 1632 [44]. Radiocarbon dates only loosely confirmed the approximate dating of the burial to between the late 15^th^ and early 17^th^ centuries (S1 Table). Besides the usual statistical outliers (2-sigma range with a probability of 95%), this was due to a plateau in the radiocarbon curve, which did not allow for the precise dating one would otherwise expect to achieve. The biological age of the deceased must also be taken into account, since radiocarbon analysis does not date the death of the individual but the period of growth during which carbon was deposited in the bones and then gradually replaced [104]. A coin minted in 1623, found near the feet of one of the individuals, was of crucial importance for the dating of the grave, since it provided a *terminus post quem* and thus acted as another piece of evidence confirming the association of the grave with the Battle of Lützen [54].

**Fig 2:**
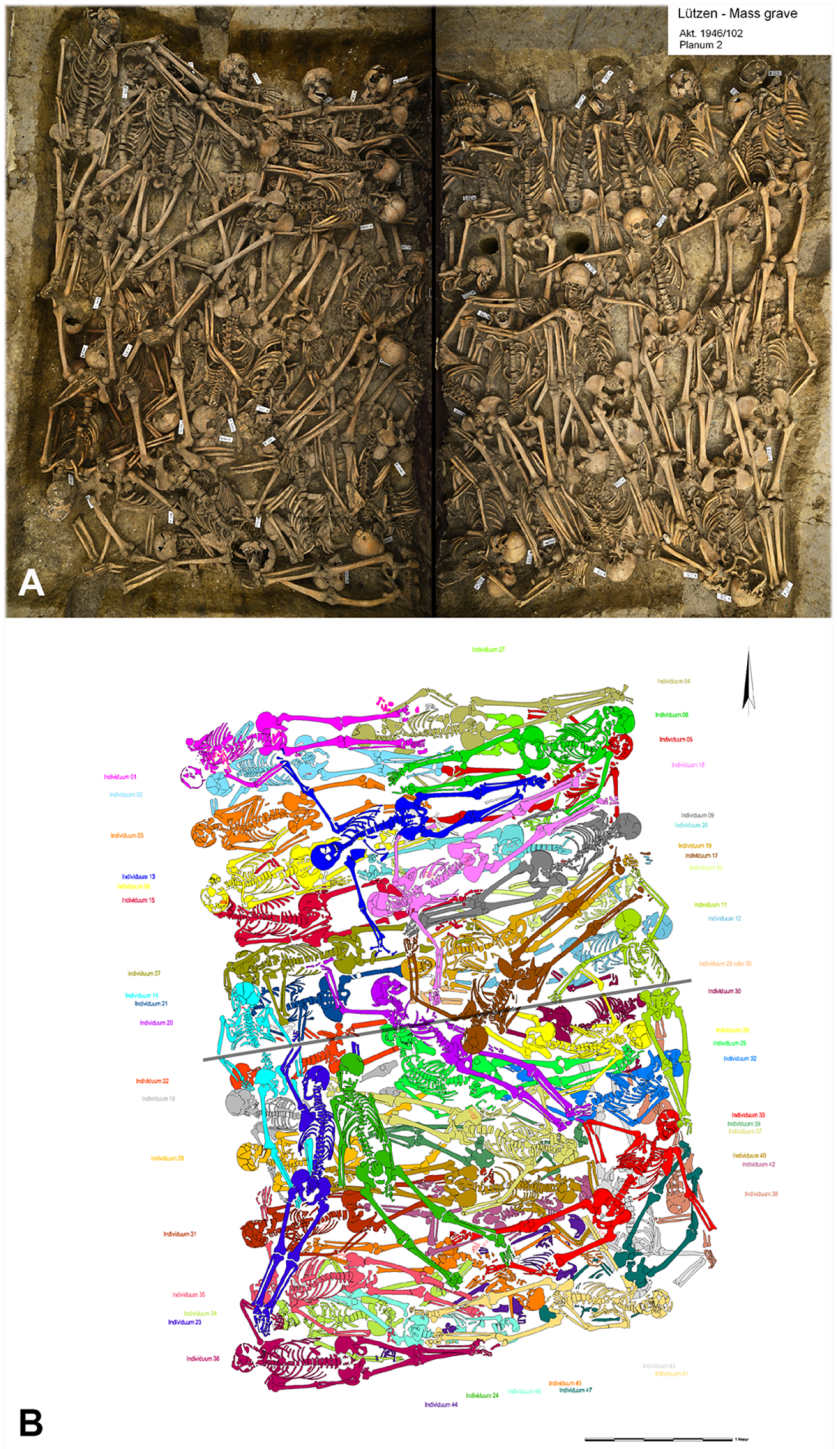
The Lützen mass grave. (A) Overall view of the mass grave and (B) graphic illustration of the skeletal remains. The skeletons have been marked in individual colours. Ten skeletons were affected by the division of the block (photos: J. Lipták, O. Schröder).

The mass grave contained the skeletal remains of a total of 47 dead (Fig 2). The bodies were found in one or two layers, with some of them lying in regular order, whilst others, however, seem to have been carelessly thrown into the pit. The state of preservation of the skeletons can be described as very good overall, which is due in large parts to the lime-rich soil. When the grave was unearthed, a small number of skeletons on the periphery (indiv. 1, 3, 6, 36, 41) were partially damaged, although this barely had an impact on the examinations because such postmortem damage to the bone can very easily be distinguished from perimortem injuries. In addition, ten skeletons (indiv. 11, 14, 17, 20, 22, 23, 25, 29, 30, 38) were affected by the division of the block, during which two to three centimetres of the skeletal elements were lost (Fig 2). Minute amounts of clothing and equipment (dress hooks, textile remnants) were found [105]. Four individuals (indiv. 7, 16, 42, 44) bore green staining on the skulls (3) and ribs (1) that can be associated with residue of non-ferrous metals. An oblong metal fragment was found lying on the sternum of a 24-30-year-old male (indiv. 31). It had left russet stains on the bones and had probably been part of his equipment. A small number of other metal fragments were found, none of which, however, have yet been identified. A ring-shaped object was found on the edge of the fill.

No distinct bite marks from carnivores were identified on the bones. Damage seen on the cancellous structures such as the joints, vertebrae and pelvises could largely be associated with postmortem processes. Some of the bones (indiv. 4 and 18) bore gnaw marks from rodents and displaced bones (e.g. the right ribcage of indiv. 13) also indicated that burrowing had taken place within the grave. This was confirmed by evidence of animal burrows and hamster bones within the grave and around its periphery.

### The basics: Sex and age

The 47 deceased buried in the mass grave were probably all men (Table 1). In eleven cases it was not possible to estimate enough of the important morphological features (cf. [59,61,62]) to give a distinct diagnosis (Table 2). However, the morphological criteria still point to male rather than female individuals. Therefore, the presence of female individuals was excluded, though this diagnosis has not yet been confirmed by molecular genetic analysis. The men had been between 15 and 50 years of age when they died, the youngest being between 14 and 16 (indiv. 32) and the oldest between 40 and 50 (indiv. 18; Table 1). The age categories listed in Table 2 were based on the mean values of the age ranges. The age distribution shows that the majority of the individuals had reached an age of 20 to 30. The average age at the time of death was calculated to have been approximately 28 years.

**Table 2:** Age distribution.

Age category (years)								
	15–20	21–25	26–30	31–40	41–50	total	mean	± SD
n individuals	5	13	13	14	2	47	27.8	6.88
%	10.6%	27.7%	27.7%	29.8%	4.3%	100%	---	---

It should be noted that the low age at death ranges in the adolescent and young adults (15-25/30 years) reflect slight differences in the degree of epi- and apophyseal union combined with other features (i.e. dentition, pubic symphysis). However, these individuals may eventually be grouped into broader ranges of 14 to 20 and 21 to 30 years age at death. More information about the age and sex profiles of the individuals is given in S2 Table.

### Trauma analysis

The skeletons exhibited a multitude of traumatic and pathological modifications, some of which could be associated with violent encounters on the battlefield, while others were linked to the living conditions that existed at the time. In addition to the data given in Tables 3 and 4, further information on estimated prevalence rates is summed up in S3 and S4 Tables.

**Table 3:** Results of the trauma analysis. Number of cranial injuries listed by location, side, and type of injury.

CRANIAL INJURIES					
Location	side	antemortem	perimortem		
			blunt	sharp	projectile*
Frontal	R	1	1	0	4
	L	3	2/1	0	3
Parietal	R	3	0	0	4
	L	5	1	0	6
	C	0	0	1	0
Occipital	R	0	0	0	2
	L	0	0/1	0	1
	C	2	0	2	0
Facial	R	0	0	1	0
	L	2	0	1	1**
	C	0	9/5	0	0
Basis	-	0	0	0	1
Total	reliable	16	13	5	22
	probable	---	7	---	2

R = right, L = left, C = central; reliable/probable;

*entry wounds;

**location of the bullet

**Table 4:** Results of the trauma analysis. Number of postcranial injuries listed by location, side, type of injury and bone elements affected.

POSTCRANIAL INJURIES			
Location	side	n	bone elements
**Antemortem**			
Upper limb	R	4	ulna, radius, metacarpus (2)
	L	4	ulna, radius, metacarpus, phalanx
Lower limb	R	5	femur (3), fibula, metatarsus
	L	5	femur (3), tibia, fibula
Torso	-	12	ribcage (5), vertebra (4), sacrum (2), pelvis
Total	reliable	30	
	probable	---	
**Perimortem: blunt**			
Upper limb	R	1	ulna
	L	3	ulna, radius, metacarpus
Lower limb	R	1	metatarsus
	L	2	femur (2)
Torso	-	1/4	ribcage/scapula (2), clavicle, ribcage
Total	reliable	8	
	probable	4	
**Perimortem: sharp**			
Upper limb	R	1	radius
	L	2	humerus, radius
Lower limb	R	4	femur (3), tibia
	L	0	---
Torso	-	4/4	scapula, vertebra (2), pelvis/scapula, pelvis
Total	reliable	11	
	probable	4	
**Perimortem: projectile**			
Upper limb	R	0/1	---/ulna
	L	0	---
Lower limb	R	3	femur, tibia (2)
	L	2	tibia (2)
Torso	-	5/1	ribcage, scapula, vertebra, pelvis (2)/sternum
Total	reliable	10	
	probable	2	

R = right, L = left; reliable/probable; (number of affected bones)

#### Antemortem trauma

**Fig 3:**
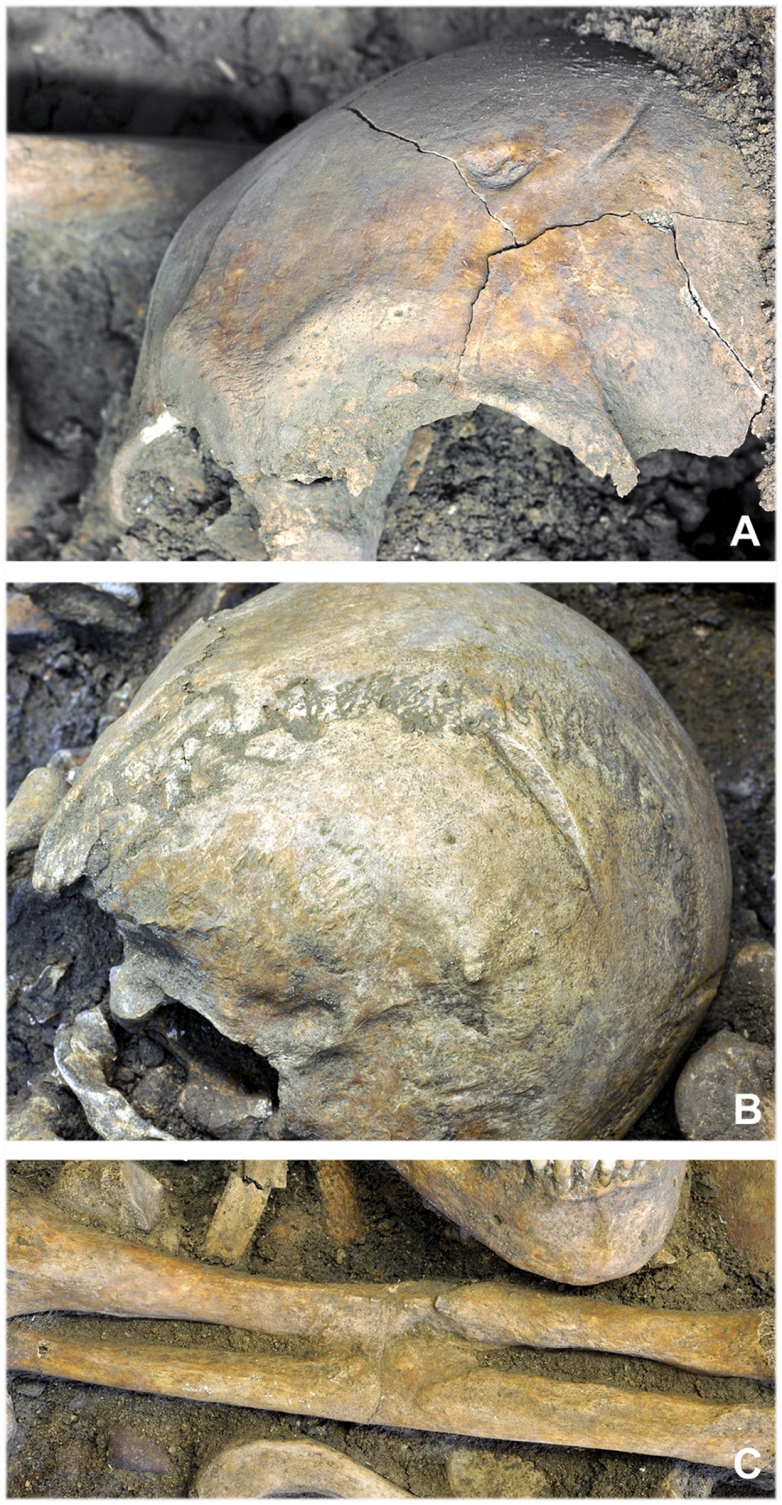
Antemortem injuries. (A) Two healed injuries from blunt and sharp force to the frontal bone of individual 46. (B) Sharp force injury to the occipital bone of individual 23 with traces of healing. (C) The injury to the left forearm of individual 18 led to the radius and ulna fusing together (photos: A. Hörentrup).

**Fig 4:**
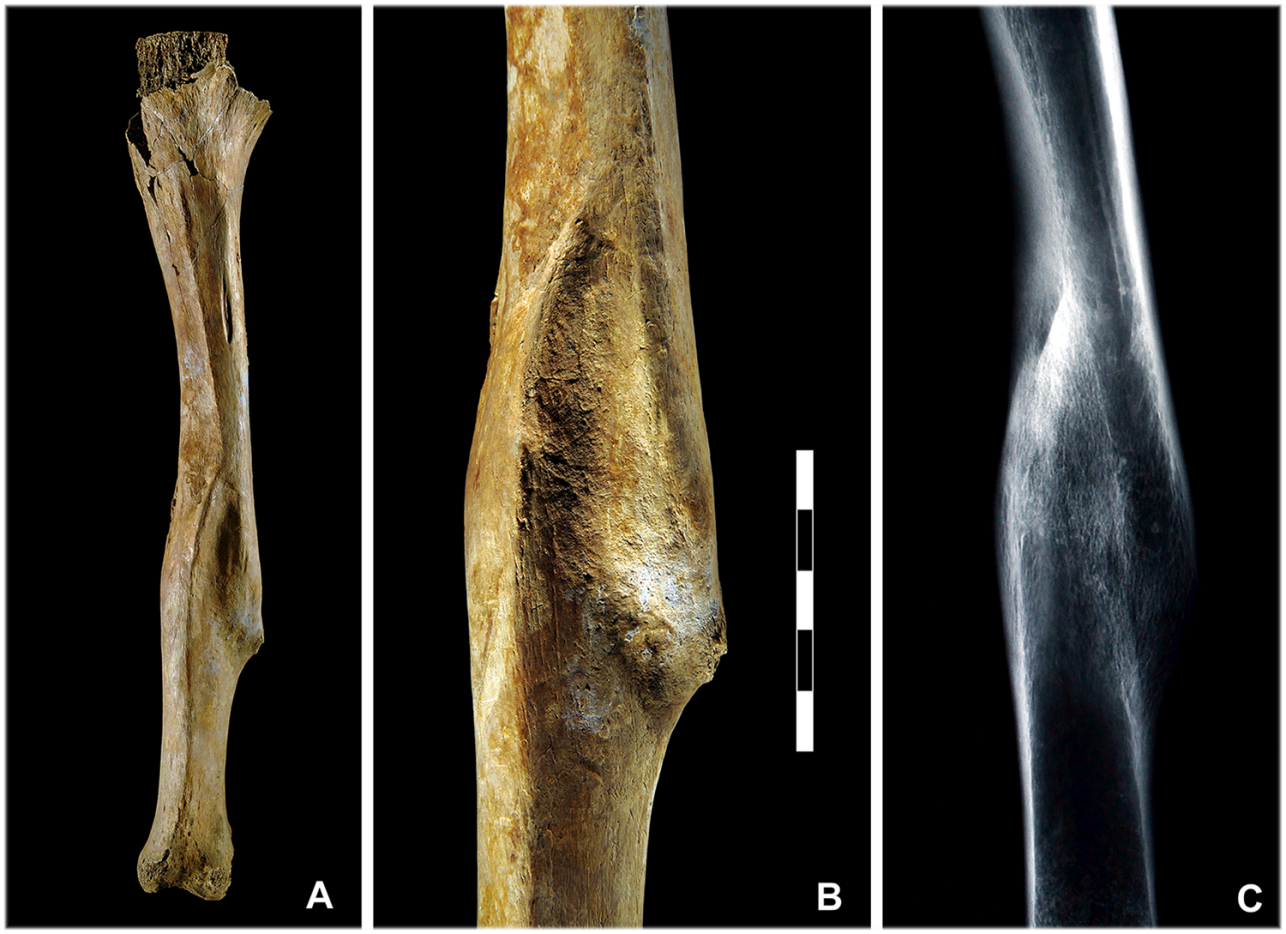
Healed long bone fracture. (A, B) The left tibia of individual 17 shows a healed fracture. (C) The fracture ends did not reunite correctly due to the clear malunion. However, the healed bone exhibits no evidence to suggest infection (photos: K. Bentele; radiograph: LDA Halle/Saale).

A total of 16 healed cranial wounds were identified in 12 of the deceased (25.5%) (Tables 1 and 3). The majority were impression fractures of the cranial vault or nasal bone fractures, which were attested to by indentations, grooves or bending (Fig 3) and were located mainly on the left frontal and parietal bones. One of the males concerned (indiv. 23) had received four sharp force cranial injuries in previous conflicts. The postcranial skeletons bore even more healed injuries, with 21 of the individuals (44.7%) exhibiting a total of 30 healed or healing bone injuries (Tables 1 and 4), including long-bone fractures (forearm, thigh, lower leg) and injuries to the hands and feet as well as fractured ribs (Figs 3 and 4).

#### Perimortem blunt force injuries

**Fig 5:**
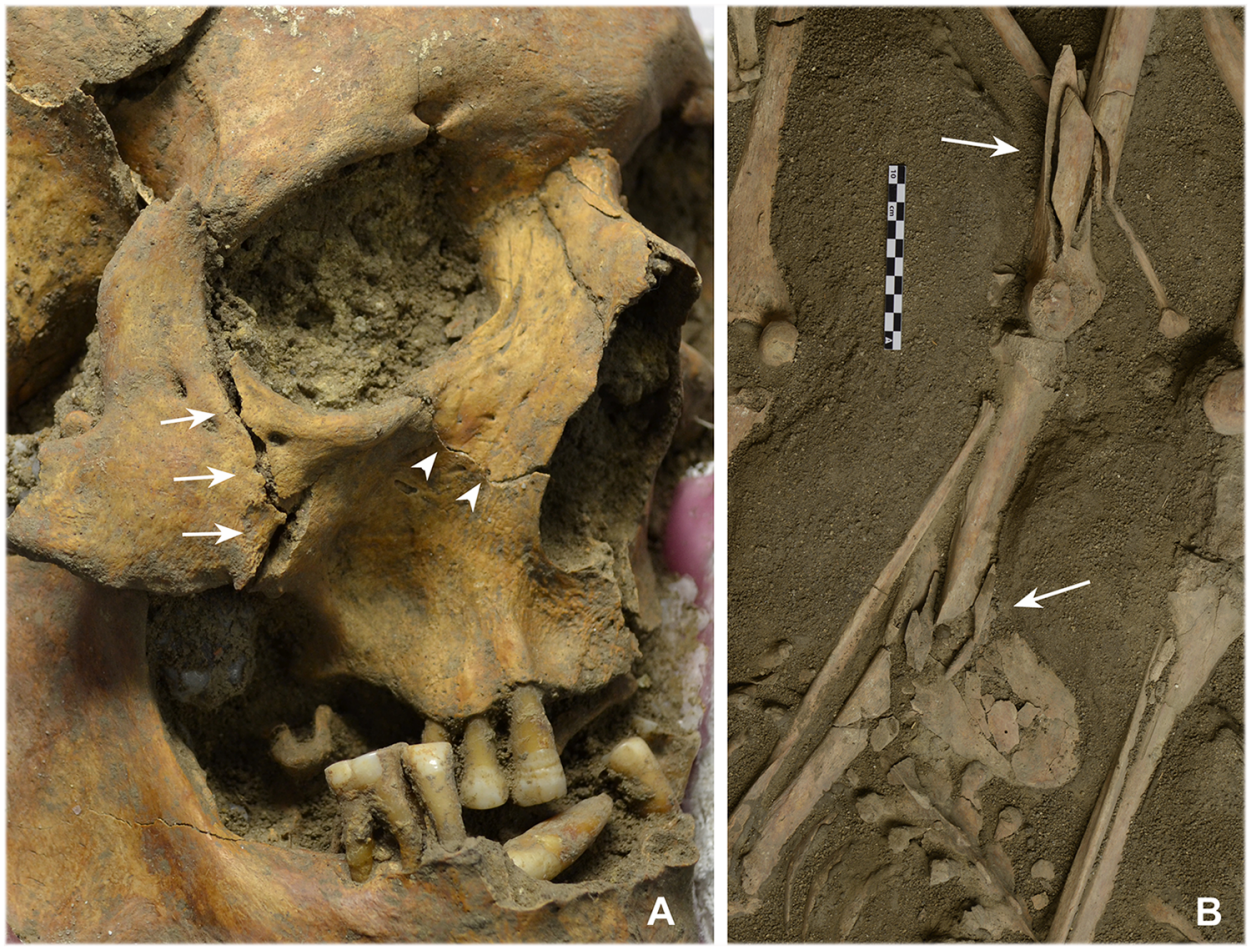
Perimortem blunt force traumata. (A) The oldest individual among the dead has fractures to the right zygomatic bone and the jawbone (indiv. 18). (B) The fracture to the right femur of individual 16 can be associated with a fall, whilst the comminuted fracture of the tibia was caused by a gunshot (photos: N. Nicklisch, J. Lipták). It is possible that the shot to the tibia provoked the fall and subsequent femur fracture.

Twelve individuals (25.5%) had 13 cranial traumata caused by blunt force (Tables 1 and 3). Most were fractures of the jaw and facial areas (Fig 5). Six men exhibited a further seven potential injuries, but these could not be interpreted beyond doubt because the bone structure had changed over the long period of deposition and similar fractures can be caused by taphonomic processes. Similar statements can be made with regard to the postcranial bones. Six men (12.8%) had eight fractures that could be explained by blunt force, whilst three others had four possible injuries (Tables 1 and 4). The main areas concerned were forearms, ribs and hands, which exhibited mostly simple two-segmented fractures such as transverse or oblique fractures. However, as shown in Fig 5B, some more complex fractures were also identified.

#### Perimortem sharp force injuries

**Fig 6:**
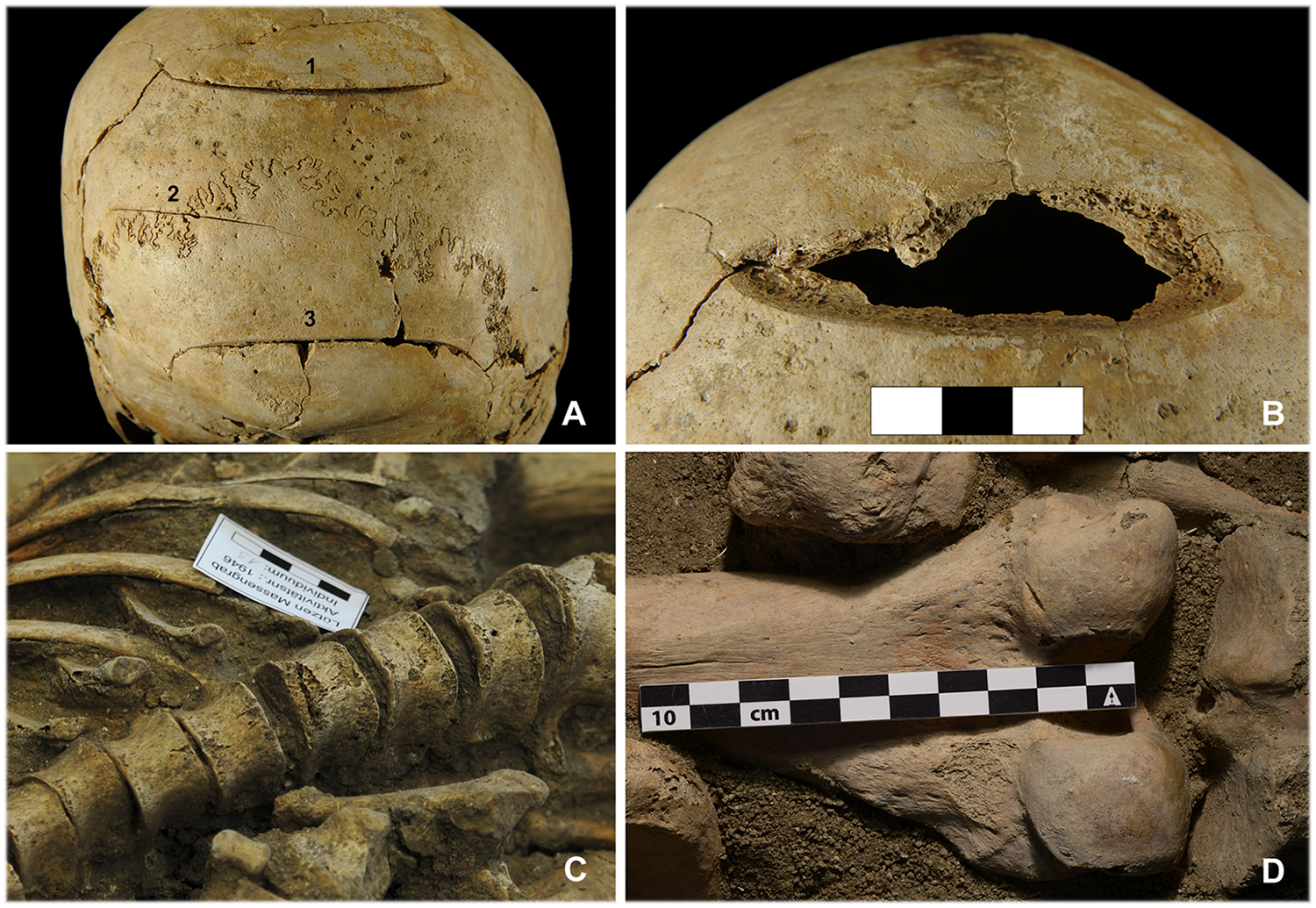
Perimortem sharp force injuries. (A) The only severe cranial injury caused by a bladed weapon was identified on individual 9. (B) Injury 1 (cf. photo A) led to the complete detachment of the bone area. (C) A penetrating injury can be seen in the anterior area of the 10^th^ thoracic vertebra of individual 13. (D) The quite inconspicuous cutting injury to the lateral condyle of the right femur of individual 7 may also have caused damage to the entheses and the popliteal artery. The latter would have resulted in severe blood loss (photos: K. Bentele, N. Nicklisch, J. Lipták).

Unhealed cranial injuries caused by bladed weapons were only identified in two of the individuals (4.2%; Tables 1 and 3). A 15-19-year-old individual (indiv. 9) had several sword or sabre cuts to the back of the head (Fig 6). The second individual, also quite a young man in his early twenties (indiv. 35) bore two cuts to the right zygomatic bone, which he probably received in a knife attack shortly before he was killed by a gunshot wound to the head. Several sharp force injuries to the postcranial skeleton were identified, most of which were cutting and stabbing injuries to the vertebrate-pelvic areas and the extremities (Fig 6). This definitely applied to seven individuals (14.9%) with a total of eleven injuries (Tables 1 and 4). Three of these men had more than one postcranial wound. Four other individuals had suspected injuries, although there were problems in establishing a definitive diagnosis with regard to the iliac wing and shoulder blade areas.

#### Perimortem projectile trauma

**Fig 7:**
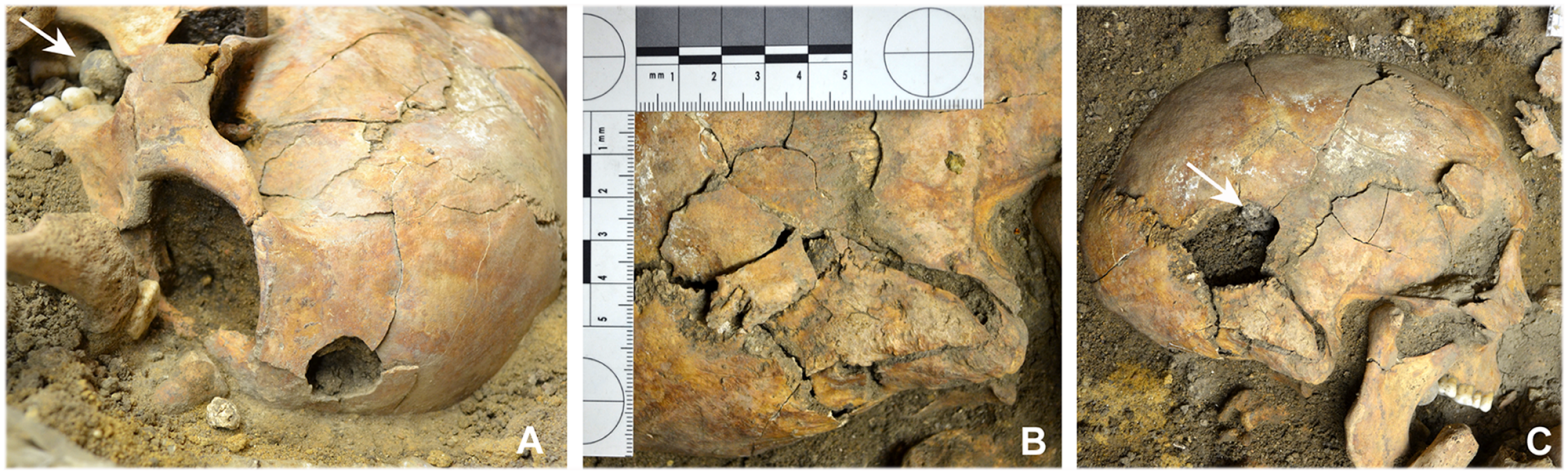
*In situ* documentation of a retained projectile. (A) Individual 22 was struck above the right forehead (entry wound). (B) The projectile crossed the cranial cavity and led to a crush fracture to the right petrous bone region and mastoid process. (C) Upon exposing the fracture area, the lead ball (from a carbine or musket) became visible (arrow). In addition, the right oral cavity contained an unfired bullet (A, arrow) (photos: N. Nicklisch).

**Fig 8:**
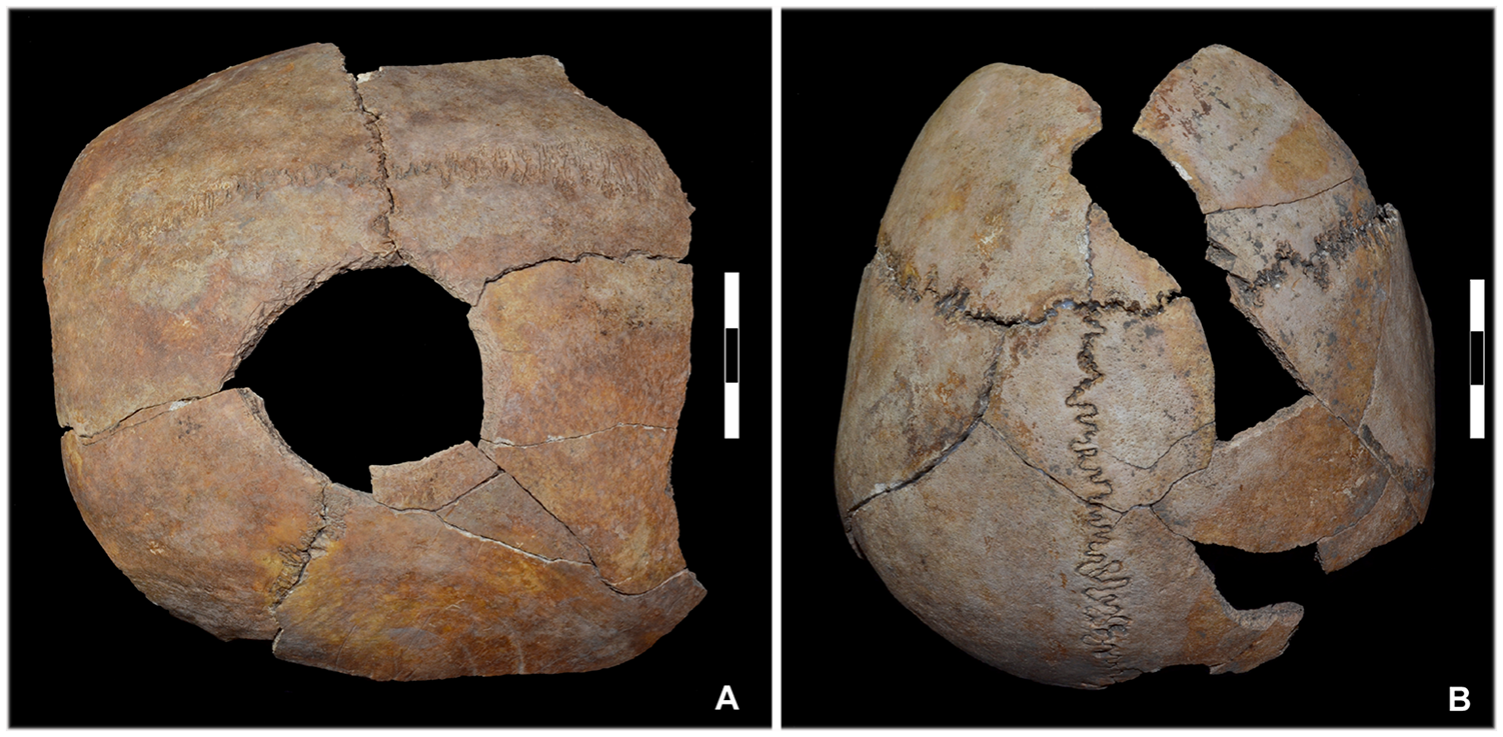
Reconstructed cranial gunshot wounds. (A) Exit wound on the left cranial vault (frontal/parietal bones) of individual 42. The entry wound can be localised on the right parietal bone. (B) Individual 35 was struck on the front cranial vault by a bullet entering at an obtuse angle (photos: N. Nicklisch).

**Fig 9:**
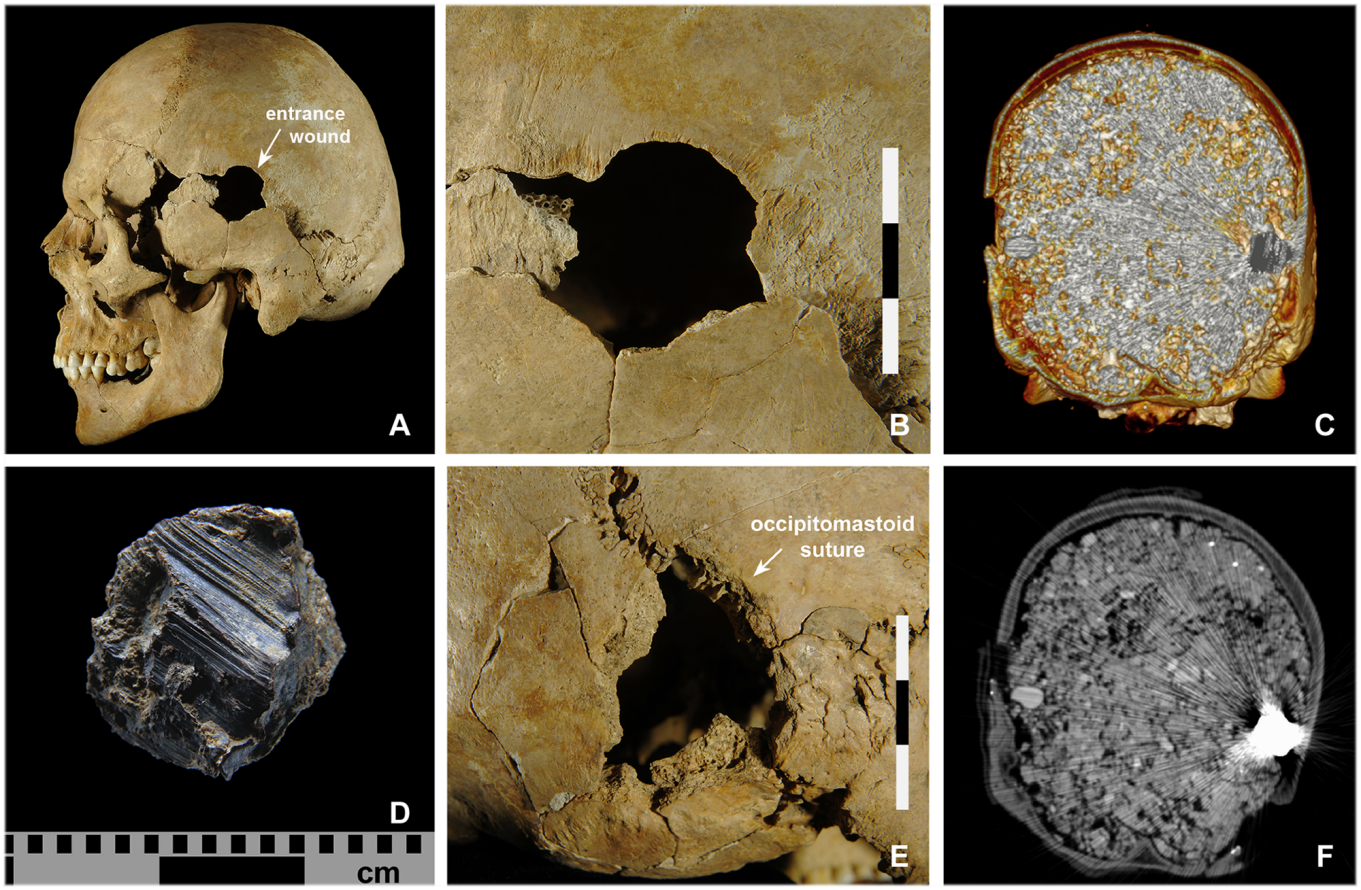
Documentation of a retained projectile upon removal of the skull. (A, B) Individual 5 was struck in the left parietal/temporal bone by a lead bullet, which (E) lodged in the right occipital bone. (D) The projectile is a musket ball. Its severe deformation suggests that it ricocheted. (C) The position of the lead bullet could be documented by CT. (F) The density and scattered radiation of the lead made it possible to distinguish clearly between the bullet and the surrounding sediment (photos: K. Bentele, N. Nicklisch, S. Brandt).

Gunshot wounds to the head were identified in 21 of the dead (44.7%) (Tables 1 and 3). A 26-35-year old male (indiv. 39) had a gunshot wound to the facial bones and neurocranium. The entry wound could only be identified on the neurocranium. Two further skeletons exhibited damage that may also have been caused by a firearm but this could not be examined in any greater detail due to the unfavourable *in situ* positions of the skulls. Eleven cases had retained projectiles, which means that the bullets were still lodged in the skulls (Fig 7). In 21 cases it was possible to identify the entry wounds (Fig 8). Several skulls (n = 17) were temporarily removed from the grave for further analysis by means of CT scans and ballistic analyses. In some of these, for instance in individual 5, the CT scans revealed the position of the lead balls that still remained in the skull (Fig 9).

**Fig 10:**
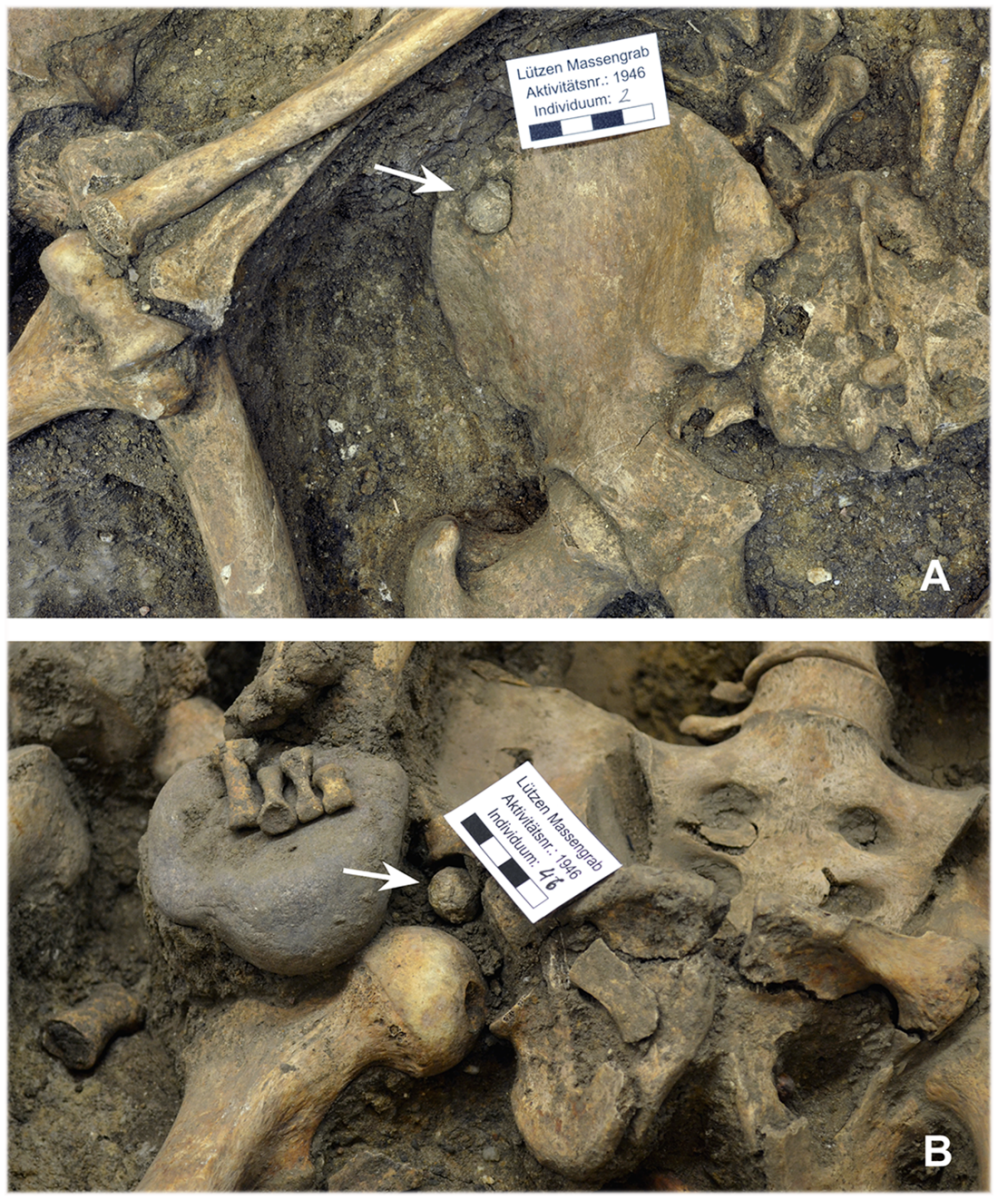
Bullets that can be associated with injuries to the postcranial skeleton. Two soldiers were struck in the hip area by carbine bullets. The projectiles remained (A) in the left ilium (indiv. 2) and (B) in the right acetabulum (indiv. 46) (photos: A. Hörentrup).

Gunshot wounds to the torso and extremities were also recorded. Eight individuals (17.0%) had a total of ten postcranial projectile injuries (Tables 1 and 4). The lead bullets were found in the hip joint, iliac bone, lumbar vertebrae and abdominal area (Fig 10). Some of the comminuted fractures of the thighs and lower legs could also be explained by projectile trauma. Two middle-aged men (indiv. 2, 16) were each hit by one bullet to the torso and another to the tibia (Fig 5). A further individual (indiv. 22) had a defect on the sternum, which may also have been caused by a gunshot. A flattened lead ball was also found on the ulna of the same individual, which may have lodged in the soft tissue without causing any significant damage to the bone.

## Discussion and conclusion

### Re-evaluating the burial process

The dead were not placed in the pit in a systematic way, starting at one end and ending at the other. Reconstructions showed that the dead had been deposited from several sides at once and no care had been taken to arrange them “in rank and file” [54]. Some of the extremities were arranged close to the body, whilst some of the dead appeared to have been thrown into the grave. In this respect the Lützen mass grave clearly differed from the regular deposition pattern observed in the mass grave at Wittstock, where researchers assume that the dead were buried under the watchful eye of the military leadership [40,48]. The civic records of Lützen include an entry made two days after the battle which states that outside help was sought to bury the dead [5]. The entry also states that the dead would be buried on the battlefield. The lack of systematic deposition is most plausibly explained by the notion that the local population helped with removing the dead bodies after the armies had moved on. Before they were placed in the pit, anything of use was taken from the bodies. The dearth of remains of clothing and equipment further supports the hypothesis that the dead were intensely plundered. The same can be said for the Wittstock mass grave. Burials from the Swedish encampment established in 1644 at Latdorf, on the other hand, show that soldiers were indeed sometimes buried with reverence wearing their uniform and with grave goods [106]. It is also supposed that special care was taken to bury the military victims of the siege of Alkmaar [53]. Studies on burial practices in multiple burials and mass graves suggest that there was a connection between the attitude of the funeral community towards the dead and how much effort was put into the burial [107]. Thus, careless treatment of the dead would suggest a neutral or even a negative attitude. It can certainly be assumed that the local population of Lützen did not have a positive attitude towards the fallen soldiers, regardless of any military affiliation. In the Thirty Years War, every battle brought destruction and deprivation for the rural population.

The lack of carnivore bite marks indicates that the bodies were either buried in a timely fashion or that the sheer mass of the dead constituted an oversupply for scavengers, resulting in the fact that not all of the bodies exhibited gnaw marks. More information was provided by the anatomical positions of the bodies (mainly of the limbs), which show that the effects of rigor mortis had already (largely) worn off by the time the bodies were placed in the grave. In view of the climatic conditions, we can therefore conclude that the dead were buried after two to four days at the earliest. In very low temperatures, rigor mortis can last for as long as two to three weeks [108,109]. Because the battle took place in November, it is possible, in principle, that rigor mortis lasted a little longer than usual. We may assume, however, that a timely burial of the dead would have been the objective in order to protect the local population from disease. The features from Lützen and also those from Wittstock are obviously in contrast to the mass grave at Alerheim, where the dead had been exposed to the summer heat for at least six weeks and decomposition was already at an advanced stage by the time the bodies were buried [41].

### Demographical aspects

More than half of the men were aged between 20 and 30 when they died, which is an age group that is known from written sources to have been targeted by recruiters [12]. A similar age distribution was also observed in the mass graves at Wittstock and Neubrandenburg [47,49]. Examinations of mass graves at Zurich and Schaffhausen, which date from 1799/1800, also identified a preferred age range between 20 and 30 years [51]. Whilst the proportion of men over the age of 35 seems to have been slightly higher in the medieval mass grave at Towton [110], the average age of the dead from the late medieval to early post-medieval mass grave at Uppsala is in a similar range to that at Lützen [52]. These demographical data clearly show that life expectancy in the army was not particularly high and that few men above the age of 40 were still able to live the high-risk life of a soldier. There is, however, evidence to suggest that veteran soldiers were appreciated for their experience and served as role models for their units [15]. Due to continuous heavy losses and the increasing shortage of soldiers, recruitment age in the latter stages of the Thirty Years War constantly decreased. This is very obviously reflected in the mass grave from the Battle of Alerheim in 1645, which contained a clearly higher proportion of 13 to 25-year-olds [41].

### Struggles in the past: Antemortem injuries

The 17^th^ century was characterised by numerous medical discoveries, including new insight into blood circulation (1628, William Harvey) and the capillary system (1661, Marcello Malpighi) [111,112]. Such scientific curiosity, however, initially had little benefit for the common soldier lying wounded on the battlefield. Anatomical knowledge about injuries and diseases was already quite extensive, although inferences and methods of treatment were little developed and still flawed by traditional teaching (humoral pathology). Examples are the works of Walter Hermann Ryff [113] and Gerard van Swieten [114] as late as the 18^th^ century. Moreover, the medical skills of most ordinary barbers or army surgeons were quite limited [111,115]. As reported by the great French surgeon Ambroise Paré (1545), patients were lucky if their bullet wounds were not treated using hot oil [111,115]. Once the surgeon managed to keep the wound clean, the chances of healing were quite good, at least in surgically uncomplicated injuries [115].

The notion that many of the men had already been involved in violent encounters before the Battle of Lützen is supported by numerous healed wounds or injuries that were in the process of healing. Most of these show no evidence of infection. Similar results were obtained by examinations of other military contexts [47,116,117,118,119]. A soldier generally ran an extremely high risk of injury, not only from interpersonal violence but also as a result of accidents occurring in everyday life or as part of his military training. Fractures of the extremities, for instance, can also be explained by a fall from a horse or carriage. Ultimately, the risk of injury would surely have been highest in combat situations. The shafts of the lower limbs are heavily mineralised regions of bone and in modern populations fractures are most common in younger adults, particularly males, due to high-energy activities. For example, femoral shaft fractures most often occur due to motor vehicle accidents or falls from a great height [120]. Two distinct fractures were identified in the mass grave at Lützen, where the bones were either not anatomically set or physical load was put on the bone too soon in the healing process (Fig 4). Since both cases (indiv. 13 and 17) involved leg bones, we may assume that mobility was limited, i.e. long marches caused problems. Since the joint status of the hip, knee and ankle was unremarkable in both men, it is possible that they were cavalrymen and were able to compensate for their handicap on horseback. However, this remains speculative. Due to the high casualty rates, it was not advantageous for the military to muster out sick or physically disabled soldiers. Moreover, the living conditions of the population had deteriorated to such an extent that many saw no option other than to join the army and remain in it for as long as possible to be able to provide for themselves and for their wives and children [12,13,15].

Healed stress-induced injuries that could be associated with physical activity were identified in two of the men: a fracture of the third metatarsal bone (indiv. 23) may have been a march fracture and two healed spinosus fractures of the cervical vertebrae (indiv. 23 and 24) may be identified as so-called clay-shovellers’ fractures. In this case such stress fractures could be explained by long marches and the digging of trenches [117,121–123]. Severe physical strain was also identified in the analysis of the Wittstock skeletons [40,47]. In their examination of German soldiers’ graves from the First World War (northern Lithuania), Jankauskas and his colleagues [117] reported on stress-induced changes to the spinal column (e.g. Schmorl’s nodes). Even more signs of physical load were identified on the skeletal remains of soldiers buried in the mass grave at Towton [124]. Some features at Lützen point to similar strain; however, the vertebrae and particularly the end plates could not be systematically examined *in situ*. Analysis of skeletal remains from a Swedish encampment established in 1644 near Latdorf indicated that they had been under severe physical strain since childhood [125]. The deceased were mainly young men and boys who, besides damage to the knees and ankles (osteochondritis dissecans), also exhibited changes to the vertebral bodies and in the shoulder area. Similar features were identified in the mass graves at Alerheim and Alkmaar, where quite a high number of joint disorders were identified in a group of individuals that also had a rather low average age [41,53]. In the case of Schmorl’s nodes caution must be exercised since a recent analysis of lower thoracic vertebrae suggests that shape and size of the vertebral components predispose to the development of these lesions [126].

Localised periosteal changes (healed lamellar bone) in the surfaces of long bones are another interesting feature. Both violent encounters and accidents can easily cause soft-tissue damage and ulceration which can lead to localised bleeding and irritation of the periosteum [127,128]. Changes that can probably be associated with such periosteal defects were identified in seven men (indiv. 2, 8, 12, 17, 18, 27, 33), mainly on the thighs and lower legs.

Evidence of deeper inflammation of the bone, possibly involving the medullary cavity (osteomyelitis), was found in some of the long bones (radius, femur, tibia) of five of the deceased. In three cases (indiv. 16, 17, 34), the infection might have been caused by soft-tissue injuries or open fractures, whilst the other two cases (indiv. 18, 23) are suspected syphilis infections [45].

### On the battlefield: Injuries inflicted during combat

The skeletons exhibited numerous injuries that can be associated with the battle and consequently with the death of the men. Projectile and blunt force traumata predominate amongst the cranial injuries, whilst the postcranial skeletons mainly bear blunt and sharp force traumata (Tables 1, 3 and 4). Some of the skeletons exhibit several of the latter. These data, however, also include doubtful cases. The *in-situ* assessment made it particularly difficult in some cases to distinguish beyond doubt between blunt force traumata and deposition-related changes. The most problematic cases were facial fractures and injuries to the finer structures of the shoulder blade and pelvic areas which, depending on the position of the bodies, may also have been caused by taphonomic processes (e.g. soil pressure and erosion, bioturbation, climate conditions) [76,129]. In some cases, stones were recorded that had caused obvious changes; fractures in the areas of bone contact (bone touching bone) must also be interpreted with caution. If such features are retrieved without the presence of an anthropologist or at least a photographic documentation, this can later cause problems during the examination in the laboratory.

Evaluating injuries caused by blunt force is generally considered difficult [76,130]. Evidence of such injuries to the cranium were mainly found on the jaw and in the facial areas, whilst on the postcranial skeleton this applied mostly to the forearms, ribs or hand and foot bones. The same skeletal regions also often exhibited healed injuries, which show that the risk of injury was highest in these areas, albeit less life-threatening. Some of the blunt force traumata can be explained by the targeted use of weapons (e.g. rifle butts or hilts) on one hand and falls from or kicks by horses on the other (e.g. [131]). Others, however, could also have occurred during removal and deposition of the dead. Injuries that one would expect to see from artillery fire, as was probably the case for instance in the mass grave at Stralsund [50], can largely be excluded here. In modern populations, similar blunt force traumata as seen at Lützen tend to occur in motor vehicle or motorcycle accidents, falls or interpersonal violence, though they are also associated with sports such as bat-and-ball games [132,133]. Clinical studies show that in homicides blunt force is most often directed at the head and neck, and trauma to the upper extremities and maxillofacial region is often described in cases of elder abuse [134,135].

The dead from the mass grave at Lützen rarely exhibited cranial injuries inflicted by bladed weapons. Cutting and penetrating injuries were somewhat more frequently found on the postcranial skeletons. Possible weapons include sabres, rapiers, knives/daggers and also halberds. The severity of the bone injuries ranged from small nicks on the surface to long deep cuts and complete detachment of entire areas of the bone (Fig 6). The latter, however, was only observed in rare cases. The fact that cutting and slashing injuries in battles from that period were common is shown by the results of the examinations carried out on the mass graves at Wittstock and Alerheim and, a century later, at Zurich and Schaffhausen [41,47,51]. Although firearms were becoming more readily available, bladed weapons were still the weapons of choice for hand-to-hand combat. Medicolegal examinations of homicide victims show that fatal sharp force traumata with bone injuries rank third after projectile and blunt force traumata, and the injuries are most often localised in the trunk region [134].

The high number of projectile injuries, which on the cranium alone amount to 45%, clearly separates the mass grave at Lützen from other, contemporary mass graves. The Wittstock assemblage also exhibited numerous projectile traumata [47], but not to the same extent as Lützen. Moreover, most of the projectile injuries at Wittstock were identified on the postcranial skeletons.

At Lützen the distribution of the projectile wounds to the skull (Table 3) suggests a perhaps surprising and quick fronto-lateral attack, which probably left the soldiers little room for evasive action. Moreover, the soldiers concerned do not appear to have had sufficient head protection. Being able to associate the ammunition with the injuries was one of the advantages of block-lifting the feature and carrying out an *in situ* analysis. The lead balls that were found in the mass grave and were still identifiable (n = 20) had probably come from pistols (1–3), muskets (5) and mainly carbines (12–14) (A. Schürger, A. Grothe unpub.). Pistols and carbines were handguns that were usually used at short distances by cavalry [95]. One of the records of the time, in fact, includes an entry that recommends that cavalrymen should aim for the enemy’s head and left side of the chest [136]. This instruction seems to have been put into practice with frightening success, at least in a small section of the Lützen battlefield.

Two of the dead still had unfired lead balls in their oral cavities (Fig 7). This is also borne out by contemporary records stating that soldiers kept bullets in their mouths to speed up the process of loading their firearms [136].

### Possible causes of death and battle reconstruction

Determinations of the cause of death based solely on bone injury patterns are problematic, because it is not the bone fracture itself that ultimately leads to a person’s death but the associated bleeding and injuries to vital organs. Cranial projectile injuries or penetrating traumata to the head inflicted by slashing weapons were usually fatal due to their impact on the brain, especially without the aid of modern intensive-care medicine [137,138]. There were certainly exceptions, but these would have been rare, particularly given the field surgery skills that existed at the time.

A highly probable cause of death could be identified for 30 of the dead soldiers (63.8%), and suspected causes of death were determined for a further eight individuals (17%) (Table 1). Only nine of the skeletons (19.1%) had no significant features that would point to a particular cause of death. In these cases, soft tissue injuries to the internal organs, particularly in the abdominal area, may have resulted in their deaths. As stated above, gunshot wounds were the predominant cause of death. More than half of the men (c. 57%) were struck by gunfire, which caused injuries that would have resulted in their deaths either instantaneously or only a short while later. Fatal injuries caused by blunt or sharp force appear to have played a minor role in this area of the battlefield or among this group of soldiers, at least as far as can be determined on the basis of the bone analysis. It is indeed possible that the men received additional heavy injuries to the soft tissue and inner organs, which did not leave any traces on the skeletons. Recent examinations show that violent attacks often lead to soft tissue injuries, only a small percentage of which, however, can be identified on the bone itself [84,139].

Actually we have only touched upon a small section of the fighting that took place on 16^th^/6^th^ November 1632. However, we may still ask if the archaeological features and the injury patterns on the skeletons might provide clues with regard to the course of the battle. The injuries identified on the majority of the dead from the mass grave at Stralsund point to artillery fire [50]. Dominant slashing injuries on the skulls from the mass grave at Alerheim are seen as evidence of an attack mounted by Bavarian cavalry on a French infantry unit [41]. In Wittstock, researchers presume that most of the dead were Swedish infantrymen who died during a confrontation with a cavalry unit [47]. But what can be said about the dead from the mass grave at Lützen? Can we reconstruct the events that occurred in the final minutes of the soldiers’ lives?

Historical records and reconstructions of the course of the battle suggest that a Swedish infantry brigade suffered a heavy defeat in the area where the mass grave was later dug. An élite unit of the Swedish army, the so-called Blue Brigade, were annihilated here in a surprise attack from the flank by an imperial cavalry unit [6,44,140]. The records speak of heavy losses. The archaeological and anthropological features support the theory that the dead from the mass grave might have been the victims of this clash. 1) The majority of the projectiles found in the grave were ammunition from hand firearms used by the cavalry. 2) More than half of the men were struck by gunfire. Injuries inflicted by bladed weapons only played a minor role. 3) The attackers primarily aimed for the head and the attack occurred mainly from the front and side.

Furthermore, initial results obtained from strontium isotope analyses exhibit a high range of geological variation (Knipper et al. in prep.) [45]. Only five of the dead yielded values that suggest probable Scandinavian origin. Provenance from Sweden or Finland can largely be excluded for approximately half of the individuals. This is what would be expected, since native Scandinavians were a minority in the army under Swedish leadership. It is important to note, that the Blue Brigade is believed to have been an élite unit that was mostly made up of German soldiers [6].

Given the confusion surrounding any battle with numerous casualties, it is plausible to assume that men from both the Swedish Protestant side and the imperial Catholic army found their final resting place in the Lützen mass grave. However, the results of our examinations allow us to surmise that perhaps not all but the majority of casualties were infantrymen of the Blue Brigade and thus soldiers serving with the Swedish army.

## Supporting information

S1 Table Results of the radiocarbon analysis. A plateau in the section of interest on the radiocarbon curve has prevented us from achieving a date of the precision one would usually expect. It should also be noted that radiocarbon analysis does not date the death of an individual but rather the period of growth during which the carbon was deposited in the bone and then gradually replaced. This means that the age of the individual must also be taken into account [104].
(PDF)

S2 Table Age and sex profiles of all individuals. (PDF)

S3 Table Observed prevalence I. Absolute numbers of reliable antemortem and perimorten injuries. Percentages relative to all bone elements or regions with at least half of the surface observable.
(PDF)

S4 Table Observed prevalence II. Absolute numbers of reliable antemortem and perimorten injuries by bone element and percentages relative to all 47 individuals.
(PDF)
