# Supplementary material for: The face of war: Trauma analysis of a mass grave from the Battle of Lützen (1632)
Source: PLoS One. 2017 May 22;12(5):e0178252. doi: 10.1371/journal.pone.0178252 (PMC5439951; doi:10.1371/journal.pone.0178252)
Supplement: S1 Table — A plateau in the section of interest on the radiocarbon curve has prevented us from achieving a date of the precision one would usually expect. It should also be noted that radiocarbon analysis does not date the death of an individual but rather the period of growth during which the carbon was deposited in the bone and then gradually replaced. This means that the age of the individual must also be taken into account [104]. (PDF) [file pone.0178252.s001.pdf]

S1 Table. Results of the radiocarbon analysis. A plateau in the section of interest on the radiocarbon curve has prevented us from achieving a date of the precision one would usually expect. It should also be noted that radiocarbon analysis does not date the death of an individual but rather the period of growth during which the carbon was deposited in the bone and then gradually replaced. This means that the age of the individual must also be taken into account [104].

ID	Lab-No.	C14 Age	±	Cal 1 sigma	Cal 2 sigma
Indiv. 8	MAMS16156	404	17	cal AD 1447-1471	cal AD 1442-1610
Indiv. 9	MAMS16157	343	21	cal AD 1491-1630	cal AD 1470-1634
Indiv. 14	MAMS16158	351	21	cal AD 1482-1625	cal AD 1460-1633
Indiv. 19	MAMS17431	369	21	cal AD 1463-1617	cal AD 1451-1630
Indiv. 28	MAMS17430	356	21	cal AD 1473-1620	cal AD 1456-1632
Indiv. 32	MAMS17429	402	17	cal AD 1448-1472	cal AD 1442-1611
Indiv. 40	MAMS17432	374	21	cal AD 1456-1615	cal AD 1449-1628
