# Supplementary material for: The face of war: Trauma analysis of a mass grave from the Battle of Lützen (1632)
Source: PLoS One. 2017 May 22;12(5):e0178252. doi: 10.1371/journal.pone.0178252 (PMC5439951; doi:10.1371/journal.pone.0178252)
Supplement: S2 Table — (PDF) [file pone.0178252.s002.pdf]

S2 Table. Age and sex profiles of all individuals.

ID	Age range (years)	Age category (years)	Age estimation	Sex	Sex determination
**I1**	26-35	31-40	epi/apophyses fused, pubic symphysis, dental wear	M	pelvis, robust long bones
**I2**	30-40	31-40	epi/apophyses fused, pubic symphysis, cranial sutures, dental wear	M	pelvis, cranium, robust long bones
**I3**	19-26	21-25	epiphyses fused, apophyses in fusion (i.e. iliac crest), molar root formation complete, dental wear	M	pelvis, cranium, robust long bones
**I4**	19-26	21-25	epiphyses fused, apophyses in fusion (i.e. iliac crest), auricular surface, molar root formation completed	M	pelvis, cranium, robust long bones
**I5**	30-40	31-40	epi/apophyses fused, cranial sutures, dental wear	M	pelvis, cranium, robust long bones
**I6**	19-26	21-25	epiphyses fused, apophyses in fusion (i.e. iliac crest), molar root formation completed, dental wear	M	pelvis, robust long bones
**I7**	24-30	26-30	epi/apophyses fused, pubic symphysis, dental wear	M	pelvis, cranium, robust long bones (e.g. femur epiphysis)
**I8**	35-45	31-40	epi/apophyses fused, pubic symphysis, cranial sutures, (dental wear > severe AMTL)	M	pelvis, cranium, robust long bones
**I9**	15-19	15-20	most epi/apophyses unfused, second molars complete, third molars in eruption	M	pelvis, cranium
**I10**	15-18	15-20	some epi/apophyses in fusion, pubic symphysis	M?	pelvis, gracile long bones
**I11**	24-30	26-30	epi/apophyses fused, pubic symphysis, cranial sutures, dental wear	M	pelvis, cranium
**I12**	30-40	31-40	epi/apophyses fused (some fusion lines still visible), cranial sutures, dental wear	M	pelvis, cranium, robust long bones
**I13**	25-30	26-30	epi/apophyses fused (except clavicle), pubic symphysis, dental wear	M	pelvis, cranium
**I14**	25-35	26-30	epi/apophyses fused, cranial sutures, dental wear	M	cranium, robust long bones, (pelvis)
**I15**	30-40	31-40	epi/apophyses fused, auricular surface, cranial sutures, dental wear	M	pelvis, robust long bones
**I16**	25-35	26-30	epi/apophyses fused (some fusion lines still visible), cranial sutures, dental wear	M	pelvis, cranium
**I17**	19-26	21-25	epiphyses fused, apophyses in fusion (iliac crest), dental wear, molar root formation completed	M	pelvis, cranium, robust long bones
**I18**	40-50	41-50	epi/apophyses fused, pubic symphysis, cranial sutures, (dental wear > severe AMTL)	M	pelvis, cranium, robust long bones
**I19**	35-45	31-40	epi/apophyses fused, pubic symphysis, dental wear	M	pelvis, cranium
**I20**	19-26	21-25	epi/apophyses fused (except iliac crest, clavicle), dental wear	M	pelvis, cranium
**I21**	24-30	26-30	epi/apophyses fused (except clavicle, some fusion lines still visible), pubic symphysis	M?	pelvis, cranium, robust long bones
**I22**	19-26	21-25	epiphyses fused, apophyses in fusion (i.e. iliac crest), dental wear, molar root formation completed	M?	cranium, (pelvis)
**I23**	24-30	26-30	epi/apophyses fused, cranial sutures, dental wear	M	pelvis, cranium, robust long bones
**I24**	16-20	15-20	epi/apophyses in fusion (acetabulum fused), third molars > root formation not completed	M	pelvis, cranium, robust long bones
**I25**	30-40	31-40	epi/apophyses fused, pubic symphysis, dental wear	M?	pelvis, cranium
**I26**	25-35	26-30	epi/apophyses fused, pubic symphysis, dental wear	M	pelvis, cranium
**I27**	30-40	31-40	epi/apophyses fused, pubic symphysis, cranial sutures, dental wear	M?	pelvis, gracile long bones
**I28**	19-26	21-25	epiphyses fused, apophyses in fusion (i.e. iliac crest), pubic symphysis	M	pelvis, cranium, robust long bones (e.g. femur epiphysis)
**I29**	19-26	21-25	epiphyses fused, apophyses in fusion (i.e. iliac crest), dental wear, molar root formation completed	M	pelvis, cranium
**I30**	18-25	15-20	epi/apophyses in fusion (femur head almost fused, iliac crest unfused)	M?	pelvis
**I31**	24-30	26-30	epi/apophyses fused, pubic symphysis, auricular surface, cranial sutures, dental wear	M	pelvis, cranium
**I32**	14-16	15-20	epi/apophyses unfused (radius + ulna prox. in fusion), second molars complete, third molars in eruption	M?	pelvis, gracile long bones
**I33**	19-26	21-25	epiphyses fused, apophyses in fusion (iliac crest), dental wear, molar root formation completed	M	pelvis, robust long bones
**I34**	24-30	26-30	epi/apophyses fused (some fusion lines still visible), dental wear	M	pelvis, cranium, robust long bones (e.g. femur epiphysis)
**I35**	19-26	21-25	epiphyses fused, apophyses in fusion (clavicle, iliac crest, ischial tuberosity)	M?	cranium, robust long bones, (pelvis)

**I36**	19-26	21-25	epiphyses fused, apophyses in fusion (i.e. iliac crest), auricular surface, pubic symphysis, dental wear	M?	cranium, pelvis, gracile long bones
**I37**	26-35	31-40	epi/apophyses fused, fusion lines visible (clavicle), pubic symphysis, auricular surface, (dental wear > AMTL, caries)	M	pelvis, cranium, robust long bones
**I38**	30-40	31-40	epi/apophyses fused, pubic symphysis, dental wear	M	pelvis, cranium
**I39**	26-35	31-40	epi/apophyses fused, fusions lines visible (clavicle), pubic symphysis, dental wear	M	pelvis, cranium, robust long bones
**I40**	30-40	31-40	epi/apophyses fused, pubic symphysis, dental wear	M	pelvis, cranium, robust long bones
**I41**	19-26	21-25	epi/apophyses in fusion (iliac crest, clavicle), dental wear	M	cranium, robust long bones (epiphyses of femur + humerus)
**I42**	40-50	41-50	epi/apophyses fused, pubic symphysis, cranial sutures, (dental wear > severe AMTL, caries)	M?	pelvis, cranium, gracile long bones
**I43**	24-30	26-30	epi/apophyses fused (except clavicle), pubic symphysis	M	cranium, robust long bones, (pelvis)
**I44**	26-35	31-40	epi/apophyses fused, pubic symphysis, dental wear	M?	pelvis
**I45**	19-26	21-25	most epi/apophyses fused, third molars > root formation not completed	M	pelvis, cranium
**I46**	24-30	26-30	epi/apophyses fused (some fusion lines still visible), molar root formation completed	M	pelvis, cranium
**I47**	24-30	26-30	epi/apophyses fused (except clavicle), dental wear	M	pelvis, cranium, robust long bones (e.g. femur epiphysis)
