# Supplementary material for: The face of war: Trauma analysis of a mass grave from the Battle of Lützen (1632)
Source: PLoS One. 2017 May 22;12(5):e0178252. doi: 10.1371/journal.pone.0178252 (PMC5439951; doi:10.1371/journal.pone.0178252)
Supplement: S3 Table — Absolute numbers of reliable antemortem and perimorten injuries. Percentages relative to all bone elements or regions with at least half of the surface observable. (PDF) [file pone.0178252.s003.pdf]

**S3 Table. Observed prevalence I.** Absolute numbers of reliable antemortem and perimorten injuries. Percentages relative to all bone elements or regions with at least half of the surface observable.

| **Bone element** | **Antemortem** | | **Perimortem injuries** | | | | | | |
|---|---|---|---|---|---|---|---|---|---|
| **Cranial** | **n** | **%** | **n blunt** | **%** | **n sharp** | **%** | **n projectile** | **%** | **n observed (> 50% of bone area)** |
| Frontal R | 1 | 3.2 | 1 | 3.2 | 0 | 0.0 | 4 | 12.9 | 31 |
| Frontal L | 3 | 7.9 | 2 | 5.3 | 0 | 0.0 | 3 | 7.9 | 38 |
| Parietal R | 3 | 10.0 | 0 | 0.0 | 0 | 0.0 | 4 | 13.3 | 30 |
| Parietal L | 5 | 13.9 | 1 | 2.8 | 0 | 0.0 | 6 | 16.7 | 36 |
| Parietal C | 0 | 0.0 | 0 | 0.0 | 1 | 3.3 | 0 | 0.0 | 30 |
| Occipital R | 0 | 0.0 | 0 | 0.0 | 0 | 0.0 | 2 | 5.1 | 39 |
| Occipital L | 0 | 0.0 | 0 | 0.0 | 0 | 0.0 | 1 | 2.4 | 42 |
| Occipital C | 2 | 5.0 | 0 | 0.0 | 2 | 5.0 | 0 | 0.0 | 40 |
| Facial R | 0 | 0.0 | 0 | 0.0 | 1 | 4.0 | 0 | 0.0 | 25 |
| Facial L | 2 | 5.7 | 0 | 0.0 | 1 | 2.9 | 1 | 2.9 | 35 |
| Facial C | 0 | 0.0 | 9 | 30.0 | 0 | 0.0 | 0 | 0.0 | 30 |
| Basis | 0 | 0.0 | 0 | 0.0 | 0 | 0.0 | 1 | 6.3 | 16 |
| Total n | 16 | | 13 | | 5 | | 22 | | |

| | **Antemortem** | | **Perimortem injuries** | | | | | | |
|---|---|---|---|---|---|---|---|---|---|
| **Postcranial** | **n** | **%** | **n blunt** | **%** | **n sharp** | **%** | **n projectile** | **%** | **n observed (> 50% of bone area)** |
| Scapula R | 0 | 0.0 | 0 | 0.0 | 1 | 4.0 | 0 | 0.0 | 25 |
| Scapula L | 0 | 0.0 | 0 | 0.0 | 0 | 0.0 | 1 | 4.3 | 23 |
| Humerus L | 0 | 0.0 | 0 | 0.0 | 1 | 2.9 | 0 | 0.0 | 35 |
| Radius R | 1 | 2.9 | 0 | 0.0 | 1 | 2.9 | 0 | 0.0 | 35 |
| Radius L | 1 | 3.6 | 1 | 3.6 | 1 | 3.6 | 0 | 0.0 | 28 |
| Ulna R | 1 | 2.8 | 1 | 2.8 | 0 | 0.0 | 0 | 0.0 | 36 |
| Ulna L | 1 | 3.3 | 1 | 3.3 | 0 | 0.0 | 0 | 0.0 | 30 |
| Metacarpal R | 2 | 5.9 | 0 | 0.0 | 0 | 0.0 | 0 | 0.0 | 34 |
| Metacarpal L | 1 | 3.4 | 1 | 3.4 | 0 | 0.0 | 0 | 0.0 | 29 |
| Phalanx L | 1 | 4.5 | 0 | 0.0 | 0 | 0.0 | 0 | 0.0 | 22 |
| Ribs R | 4 | 18.2 | 0 | 0.0 | 0 | 0.0 | 1 | 4.5 | 22 |
| Ribs L | 1 | 3.8 | 1 | 3.8 | 0 | 0.0 | 0 | 0.0 | 26 |
| Cervical spine | 1 | 4.2 | 0 | 0.0 | 0 | 0.0 | 0 | 0.0 | 24 |
| Thoracic spine | 1 | 3.1 | 0 | 0.0 | 1 | 3.1 | 0 | 0.0 | 32 |
| Lumbar spine | 2 | 5.3 | 0 | 0.0 | 1 | 2.6 | 1 | 2.6 | 38 |
| Sacrum | 2 | 6.7 | 0 | 0.0 | 0 | 0.0 | 0 | 0.0 | 30 |
| Pelvis R | 0 | 0.0 | 0 | 0.0 | 0 | 0.0 | 1 | 2.6 | 39 |
| Pelvis L | 1 | 2.7 | 0 | 0.0 | 1 | 2.7 | 1 | 2.7 | 37 |
| Femur R | 3 | 7.0 | 0 | 0.0 | 3 | 7.0 | 1 | 2.3 | 43 |
| Femur L | 3 | 7.1 | 2 | 4.8 | 0 | 0.0 | 0 | 0.0 | 42 |
| Tibia R | 0 | 0.0 | 0 | 0.0 | 1 | 2.4 | 2 | 4.9 | 41 |
| Tibia L | 1 | 2.4 | 0 | 0.0 | 0 | 0.0 | 2 | 4.8 | 42 |
| Fibula R | 1 | 2.6 | 0 | 0.0 | 0 | 0.0 | 0 | 0.0 | 38 |
| Fibula L | 1 | 3.0 | 0 | 0.0 | 0 | 0.0 | 0 | 0.0 | 33 |
| Metatarsal R | 1 | 4.2 | 1 | 4.2 | 0 | 0.0 | 0 | 0.0 | 24 |
| Total n | 30 | | 8 | | 11 | | 10 | | |

n.d.=not determinable; R=right, L=left, C=central
