# Supplementary material for: The face of war: Trauma analysis of a mass grave from the Battle of Lützen (1632)
Source: PLoS One. 2017 May 22;12(5):e0178252. doi: 10.1371/journal.pone.0178252 (PMC5439951; doi:10.1371/journal.pone.0178252)
Supplement: S4 Table — Absolute numbers of reliable antemortem and perimorten injuries by bone element and percentages relative to all 47 individuals. (PDF) [file pone.0178252.s004.pdf]

S4 Table. Observed prevalence II. Absolute numbers of reliable antemortem and perimorten injuries by bone element and percentages relative to all 47 individuals.

						Min. prevalence = uncorrected crude rate*				Max. prevalence = uncorrected upper rate*			
**Bone element**	**Antemortem**	**Perimortem injuries**			**Missing data**	**Antemortem**	**Perimortem**			**Antemortem**	**Perimortem**		
**Cranial**	**n**	**n blunt**	**n sharp**	**n projectile**		**%**	**blunt %**	**sharp %**	**projectile %**	**%**	**blunt %**	**sharp %**	**projectile %**
Frontal R	1	1	0	4	16	2.1	2.1	0.0	8.5	35.0	35.0	34.0	38.0
Frontal L	3	2	0	3	9	6.4	4.3	0.0	6.4	22.1	21.1	19.1	22.1
Parietal R	3	0	0	4	17	6.4	0.0	0.0	8.5	39.2	36.2	36.2	40.2
Parietal L	5	1	0	6	11	10.6	2.1	0.0	12.8	28.4	24.4	23.4	29.4
Parietal C	0	0	1	0	17	0.0	0.0	2.1	0.0	36.2	36.2	37.2	36.2
Occipital R	0	0	0	2	8	0.0	0.0	0.0	4.3	17.0	17.0	17.0	19.0
Occipital L	0	0	0	1	5	0.0	0.0	0.0	2.1	10.6	10.6	10.6	11.6
Occipital C	2	0	2	0	7	4.3	0.0	4.3	0.0	16.9	14.9	16.9	14.9
Facial R	0	0	1	0	22	0.0	0.0	2.1	0.0	46.8	46.8	47.8	46.8
Facial L	2	0	1	1	12	4.3	0.0	2.1	2.1	27.5	25.5	26.5	26.5
Facial C	0	9	0	0	17	0.0	19.1	0.0	0.0	36.2	45.2	36.2	36.2
Basis	0	0	0	1	31	0.0	0.0	0.0	2.1	66.0	66.0	66.0	67.0
Total n	16	13	5	22	---	---	---	---	---	---	---	---	---

						Min. prevalence = uncorrected crude rate				Max. prevalence = uncorrected upper rate			
	**Antemortem**	**Perimortem injuries**			**Missing data**	**Antemortem**	**Perimortem**			**Antemortem**	**Perimortem**		
**Postcranial**	**n**	**n blunt**	**n sharp**	**n projectile**		**%**	**blunt %**	**sharp %**	**projectile %**	**%**	**blunt %**	**sharp %**	**projectile %**
Scapula R	0	0	1	0	22	0.0	0.0	2.1	0.0	46.8	46.8	47.8	46.8
Scapula L	0	0	0	1	24	0.0	0.0	0.0	2.1	51.1	51.1	51.1	52.1
Humerus L	0	0	1	0	12	0.0	0.0	2.1	0.0	25.5	25.5	26.5	25.5
Radius R	1	0	1	0	12	2.1	0.0	2.1	0.0	26.5	25.5	26.5	25.5
Radius L	1	1	1	0	19	2.1	2.1	2.1	0.0	41.4	41.4	41.4	40.4
Ulna R	1	1	0	0	11	2.1	2.1	0.0	0.0	24.4	24.4	23.4	23.4
Ulna L	1	1	0	0	17	2.1	2.1	0.0	0.0	37.2	37.2	36.2	36.2
Metacarpal R	2	0	0	0	13	4.3	0.0	0.0	0.0	29.7	27.7	27.7	27.7
Metacarpal L	1	1	0	0	18	2.1	2.1	0.0	0.0	39.3	39.3	38.3	38.3
Phalanx L	1	0	0	0	25	2.1	0.0	0.0	0.0	54.2	53.2	53.2	53.2
Ribs R	4	0	0	1	25	8.5	0.0	0.0	2.1	57.2	53.2	53.2	54.2
Ribs L	1	1	0	0	21	2.1	2.1	0.0	0.0	45.7	45.7	44.7	44.7
Cervical spine	1	0	0	0	23	2.1	0.0	0.0	0.0	49.9	48.9	48.9	48.9
Thoracic spine	1	0	1	0	15	2.1	0.0	2.1	0.0	32.9	31.9	32.9	31.9
Lumbar spine	2	0	1	1	9	4.3	0.0	2.1	2.1	21.1	19.1	20.1	20.1
Sacrum	2	0	0	0	17	4.3	0.0	0.0	0.0	38.2	36.2	36.2	36.2
Pelvis R	0	0	0	1	8	0.0	0.0	0.0	2.1	17.0	17.0	17.0	18.0
Pelvis L	1	0	1	1	10	2.1	0.0	2.1	2.1	22.3	21.3	22.3	22.3
Femur R	3	0	3	1	4	6.4	0.0	6.4	2.1	11.5	8.5	11.5	9.5
Femur L	3	2	0	0	5	6.4	4.3	0.0	0.0	13.6	12.6	10.6	10.6
Tibia R	0	0	1	2	6	0.0	0.0	2.1	4.3	12.8	12.8	13.8	14.8
Tibia L	1	0	0	2	5	2.1	0.0	0.0	4.3	11.6	10.6	10.6	12.6
Fibula R	1	0	0	0	9	2.1	0.0	0.0	0.0	20.1	19.1	19.1	19.1
Fibula L	1	0	0	0	14	2.1	0.0	0.0	0.0	30.8	29.8	29.8	29.8
Metatarsal R	1	1	0	0	23	2.1	2.1	0.0	0.0	49.9	49.9	48.9	48.9
Total n	30	8	11	10	---	---	---	---	---	---	---	---	---

*according to Waldron (1991)

Waldron T. Rates for the job. Measures of the disease frequency in paleopathology. Int J Osteoarchaeol. 1991; 1: 17–25.
